# RELAXATION INTERVENTIONS IN NEUROLOGICAL REHABILITATION: A SCOPING REVIEW

**DOI:** 10.2340/jrm.v58.45972

**Published:** 2026-10-08

**Authors:** Pia RINGS, Juliana DZIURLA, Tine VAN DAMME, Kaat ALAERTS, Christina GROLL, Dörte ZIETZ

**Affiliations:** 1 Department of Nursing, Midwifery and Therapeutic Sciences, Bochum University of Applied Sciences, Bochum, Germany; 2 Department of Rehabilitation Sciences, KU Leuven, Leuven; 3 University Psychiatric Center KU Leuven, KU Leuven, Leuven, Belgium

**Keywords:** mind–body therapies, multiple sclerosis, neurological rehabilitation, relaxation therapy, stroke, scoping review

## Abstract

**Objective:**

To offer a comprehensive synthesis that maps current research on relaxation interventions in neurological rehabilitation settings.

**Methods:**

The review was registered (DOI: 10.17605/OSF.IO/QD4TM) and followed the Joanna Briggs Institute framework. Six databases were searched from inception to August 2025. Eligible sources examined interventions aiming to induce a physiological relaxation response within neurological rehabilitation contexts. Two reviewers independently screened records and appraised study quality.

**Results:**

63 sources were included. The findings reveal a heterogeneous evidence landscape, within which several patterns emerge. Relaxation interventions have most frequently been studied in people with multiple sclerosis and stroke. They are generally reported as feasible and well accepted, but often adapted to specific functional needs and preferences. Outcomes were primarily assessed at the level of body structure and function domain, particularly psychological functioning. Key gaps in the evidence base include limited research into less prevalent conditions, few qualitative designs and comparative effectiveness studies, as well as limited assessment of outcomes relating to perceived and physiological stress, activities, and participation.

**Conclusion:**

By mapping the existing literature on relaxation interventions in neurological rehabilitation settings, the review offers a structured synthesis of the field and informs both clinical decision-making and the prioritization of future research.

## LAY ABSTRACT

People with neurological conditions experience increased stress, and relaxation interventions may help to decrease this. Until now, there has been no overview of the current knowledge on the use of relaxation interventions in neurological rehabilitation. We therefore summarized existing studies on the topic. We found that most studies included people with multiple sclerosis or stroke, while other conditions were represented less or not at all. Many of the relaxation interventions were tailored to individual needs, and no single technique has been shown to work better than others. Studies mainly looked at emotional effects, while important aspects of neurological rehabilitation such as everyday activities and participation in life were rarely considered. Overall, this review helps clinicians to see quickly what has been studied for their patient group, so they can purposefully select and adapt their relaxation interventions. Simultaneously, it highlights important gaps in research and shows what future studies are needed.

Individuals living with neurological conditions commonly experience heightened levels of psychological and physiological stress (1, 2). Stress is defined as a state in which intrinsic or extrinsic stimuli threaten the body’s homeostasis, triggering adaptive responses that can be harmful when prolonged (3, 4). Psychological stressors in neurological populations include uncertainty concerning prognosis, loss of independence, or social isolation (5–7). They can contribute to the development of mental illnesses such as anxiety or depression, which are prevalent in these populations (8–11). Also, physiologically, neurological conditions impose stress by disrupting homeostasis to a large extent. This activates the physiological stress response, characterized by sympathetic arousal and stimulation of the hypothalamic–pituitary–adrenal (HPA) axis, leading to changes across cardiovascular, respiratory, endocrine, immune, musculoskeletal, and nervous systems (12). While these responses are adaptive in the short term, persistent activation promotes autonomic imbalance, neuroinflammation, and cardiovascular strain (13–15), potentially exacerbating neurological damage and impairing recovery (1, 4, 16). For example, heightened stress among stroke survivors is associated with poorer functional outcomes, slower recovery, and an increased risk of long-term disability (17–19). In people with multiple sclerosis (MS), stress negatively impacts inflammatory activity and disease progression (20), while disease progression leads to higher psychological stress (21, 22). Managing stress in populations with neurological conditions is therefore critical for optimizing rehabilitation outcomes.

Relaxation interventions are designed to actively induce a physiological state of relaxation to change physiological and psychological responses to stress (23). This intended physiological response is characterized by a shift towards dominance of the parasympathetic branch of the autonomic nervous system (24). Structured relaxation interventions include the Benson relaxation technique (24), progressive muscle relaxation (PMR) (25), autogenic training (26), diaphragmatic breathing (DB) (27), guided imagery (GI) (28), and biofeedback interventions (29), among others. Notably, all relaxation interventions emphasize the active induction of the physiological relaxation response. They can therefore be distinguished conceptually from mindfulness-, movement- or leisure-based activities, for which relaxation may be a side effect but is not the primary goal.

The physiological relaxation response, mediated via the autonomic nervous system, is associated with multiple physiological changes. The modulation of the HPA axis results in reduced cortisol secretion (30, 31), as well as improved cardiovascular (24, 32, 33) and respiratory function (34). Psychologically, the relaxation response is associated with reduced levels of anxiety (35, 36), depression (36), and psychological distress (36, 37). Despite this anticipated capacity to influence multiple domains commonly affected by neurological conditions, the potential of relaxation interventions for stress management in neurological populations remains relatively underexplored. While pooled evidence supports the integration of mindfulness- and movement-based practices into neurological rehabilitation (38–42) and one systematic review has examined relaxation and related therapies in people with MS, no comparable synthesis of studies on relaxation interventions in the broader neurological rehabilitation context exists. This lack of structured evidence leaves clinicians with limited guidance for implementation, and researchers with little direction for future inquiry. To address this gap, the present scoping review synthesizes the available literature on relaxation interventions in neurological rehabilitation. The primary research question is: What is the current state of research on relaxation interventions in neurological rehabilitation? Specifically, populations, intervention types, settings, and assessed outcomes are mapped. Together, this aims to clarify the current evidence base and identify key research gaps.

## METHODS

### Protocol and registration

The review was registered on the Open Science Frame-work (registration DOI: 10.17605/OSF.IO/QD4TM). It was conducted in accordance with the Joanna Briggs Institute methodology for scoping reviews (43) and reported following the PRISMA-ScR guideline (44).

### Eligibility criteria

Eligibility criteria were defined *a priori* using the Population–Concept–Context framework (43). The population of interest was individuals with a formal diagnosis of any neurological condition. No neurological conditions were excluded based on the diagnosis alone. However, studies were eligible only if they investigated the respective neurological condition within a neurological rehabilitation context, including acute, subacute, long-term inpatient, outpatient, or community-based neurological rehabilitation delivered by multidisciplinary teams. Consequently, studies on conditions primarily managed outside rehabilitation pathways, such as migraine or headache disorders, were excluded when they did not meet the context criterion. Studies with mixed populations were included if data for the neurological subgroup were extractable or comprised over 80%. The concept focused on interventions explicitly aimed to induce a physiological relaxation response. Studies without a clear reference to relaxation were excluded, as were mixed interventions, if less than 80% of components targeted relaxation. Biofeedback interventions were included only when the protocol clearly aimed to facilitate relaxation, for example through combination with a relaxation intervention or through instructions for deep, paced breathing. Biofeedback protocols targeting general self-regulation without a clear relaxation aim, or solely local muscle relaxation, were excluded. All study designs were eligible except cross-sectional studies, guidelines, comments, books, protocols, retracted articles, inaccessible full texts, and publications in languages other than English, German, or Dutch. Detailed eligibility criteria are listed in Appendix S1, Table SI.

### Search strategy

A comprehensive search strategy was developed with librarian support using keywords and synonyms for “neurological condition” and “relaxation therapy”, including condition and interventions names. The initial strategy was designed for PubMed and adapted for other databases. Grey literature was searched using simplified terms. Keywords, MeSh terms, and full search strings can be found in Appendix S2, Table SII. Searches were conducted in PubMed, Embase, AMED, CINAHL, PsycINFO, and Web of Science from inception to November 2024, with an update on 13 August 2025. Grey literature was retrieved from ClinicalTrials.gov and the ProQuest™ Dissertations & Theses Citation Index. Reference lists of relevant sources were hand-searched.

### Selection process

Two reviewers (PR, JD) independently screened titles/abstracts and full texts. Before each screening stage, consistency checks were conducted using random samples generated by CADIMA (45) from the respective set of records. Discrepancies were resolved through discussion between the two reviewers. Authors were contacted when essential information was missing. CADIMA also supported workflow management and duplicate detection.

### Data extraction and synthesis of results

A data charting form was piloted by two reviewers (PR, JD) to refine the pre-specified extraction items and promote consistent terminology. Data extraction was then performed by one reviewer (PR). Items included study details (title, authors, year, location, design, key findings), participant characteristics (diagnosis, sample size, mean age, dropout rates), intervention characteristics (technique, duration, frequency, delivery method, setting), outcomes, and assessment methods. Sources were grouped by population, and outcomes categorized on the International Classification of Functioning, Disability and Health (ICF) domains (46). Results were reported descriptively, focusing on studies that examined relaxation as the experimental condition, rather than the control group. Sources referring to the same primary dataset were merged to avoid duplication.

### Quality assessment

Although optional in scoping reviews, methodological appraisal was conducted to inform future research. Two reviewers (PR, JD) independently assessed study quality using the corresponding JBI Critical Appraisal Tool (47–52). As no JBI tool exists for mixed-methods studies, the Mixed Methods Appraisal Tool (53) was applied. Discrepancies were resolved through discussion. Overall quality was rated as high (≥ 80%), moderate (60–79%), or low (< 60%) based on the proportion of criteria met. Detailed appraisal results for each study are provided in Appendix S4; Table SIV. As different appraisal tools were used, the number and type of criteria varied across studies. Therefore, the quality assessment should be interpreted as a study-specific summary of methodological quality rather than as a directly comparable measure across different study designs.

## RESULTS

### Study selection

The search identified 6,243 sources. 63 studies, referring to 59 primary data sets, met the inclusion criteria. Study selection is shown in Fig. 1. Inter-rater agreement was high for title/abstract (κ = 0.81; *n* = 624), and full-text screening (κ = 0.88; *n* = 40). Authors were contacted in 3 cases for missing information on intervention details, with 1 response received.

**Fig. 1:**
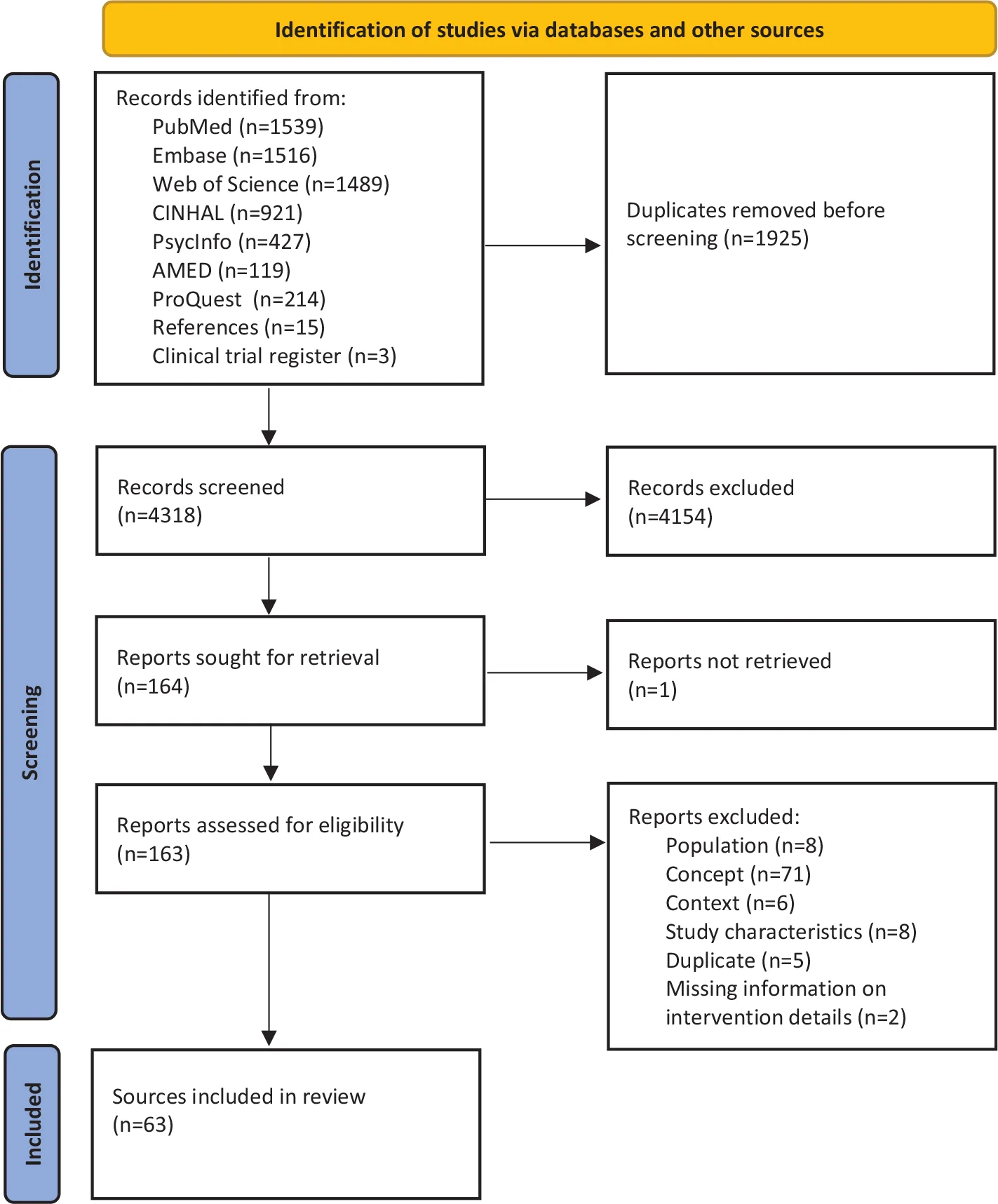
PRISMA flow diagram of study selection.

### General study characteristics

The combined sample size was 2,526 participants. Studies were conducted across various regions, mainly in Asia (*n* = 21) (54–74) and Europe (*n* = 19) (67, 75–92). Publication years ranged from 1977 to 2025, with most studies (*n* = 36) (54–57, 59–67, 71, 72, 76–78, 80, 83, 84, 86, 87, 91–102) published in the last decade. Methodological designs included randomized controlled trials (RCTs) (*n* = 28) (55–57, 61–65, 67–69, 71, 72, 75, 76, 78, 79, 81, 86, 87, 89, 90, 93, 100, 102–104, 112), pilot RCTs (*n* = 5) (80, 88, 95, 96, 105), quasi-experimental trials (*n* = 9) (54, 59, 60, 73, 74, 83, 85, 106, 107), pilot quasi-experimental trials (*n* = 2) (58, 94), mixed-methods trials (*n* = 2) (91, 92), pilot mixed-methods trials (*n* = 3) (84, 98, 108), case series (*n* = 7) (66, 70, 97, 99, 109–111), one case report (77), one qualitative co-design study (101), and a qualitative programme evaluation following an RCT (82). One study (60) was labelled as cross-sectional in its title, but the methods section described a non-randomized quasi-experimental pre–post design. Therefore, this study was classified according to its actual design as a quasi-experimental longitudinal intervention study. Populations included individuals with MS (*n* = 33) (56, 57, 59–65, 68–76, 81, 84–87, 89, 90, 93, 96, 102–106, 112), stroke (*n* = 19) (54, 55, 66, 67, 77–79, 80, 82, 88, 91, 92, 99, 101, 107, 113–116), spinal cord injury (*n* = 6) (94, 95, 97, 100, 108 109), cerebral palsy (*n* = 3) (98, 110, 111), Parkinson’s disease (*n* = 1) (58), and one mixed group (83). Settings comprised outpatient rehabilitation (*n* = 25) (56, 58–61, 64, 65, 67, 68, 71, 73, 74, 76, 81, 87–90, 93, 102, 104, 106, 108, 109, 112), community-based environments (*n* = 14) (70, 72, 80, 82, 83, 92, 95, 96, 110, 111, 105, 97, 99, 101), inpatient rehabilitation (*n* = 13) (54, 55, 57, 62, 63, 66, 77–79, 85, 91, 94, 107), one laboratory (100), and six studies (69, 75, 84, 86, 98, 103) with unclear settings.

Full details on study designs, participant characteristics, intervention types, outcomes, and quality ratings for studies investigating relaxation interventions as experimental condition are available in Table I. Information on studies with relaxation interventions as control condition are available in Appendix S3, Table SIII. Fig. 2 summarizes all investigated outcomes and their distribution by condition.

**Table I:** Characteristics of included studies with relaxation as experimental condition

Main author and year	Study design	Participants *n*, age (SD), inclusion criteria	Interventions Type, setting, length, frequency, duration	Outcomes (assessments)				Quality rating
				Body functions/structure	Activities/participation	Environmental/personal factors	Feasibility/experiences	
**MS**								
Garis et al. (2023)	RCT	*n* = 24 Age = 46 ± 13	HR- and RR-biofeedback (+DB vs +PMR) Individual Unclear, 2 sessions, 1 day	+ Fatigue (VAS) + HRV	-	-	-	Moderate
Beitollahi et al. (2022)	RCT	*n* = 60 Age = 39 ± 10 FFS < 6	GI (vs waiting list) Self-practice 5 min, 6/week, 12 weeks	+ Fatigue (FFS) + Mood (POMS)	+ Stigma (RSS-MS)	-	-	Low
Kesik et al. (2022)	RCT	*n* = 85 Age = 35 ± 11 EDSS ≤ 5.5	(1) PMR (2) BRT (vs education) Group & self-practice 20–25 min, 1 session & 1/day, 12 weeks	+ Fatigue (FSS) + Pain (VAS) + Kinesiophobia (TSK)	-	-	Compliance (phone calls)	Low
Javdan et al. (2021)	Quasi-experimental	*n* = 76 Age = 36 ± 8	PMR (vs no intervention) Group & self-practice Unclear, 4 sessions & 1/day, 8 weeks	+ Fatigue (FSS)	+ ADLs & iADLs (questionnaire)	-	-	Low
Saifan et al. (2021)	Quasi-experimental	*n* = 105 Age = 33 ± 6	BRT (vs no intervention) Self-practice 10 min, 2/day, 8 weeks	+ Depression, Anxiety, Stress (DASS-21)	-	-	-	Moderate
Mirhosseini et al. (2021)	RCT	*n* = 60 Age = 34 ± 4 EDSS < 5	BRT (vs TAU) Self-practice, group & telehealth 20 min, 2/day, 8 weeks	+ General health (GHQ-28)	-	-	-	Low
Akbari et al. (2020)	RCT	*n* = 60 Age = 36 ± 10 EDSS ≤ 4.5	PMR+BRT (vs TAU) Group, 60 min, 2/week, 4 weeks	+ Depression, Anxiety & Stress (DASS-21)	-	-	-	Moderate
Mirhosseini et al. (2019)	RCT	*n* = 60 Age = 34 ± 3 EDSS 1-5	BRT (vs TAU) Self-practice, group & telehealth 20 min, 2/day, 4 weeks	+ Fatigue (BFI)	-	-	-	Low
Case et al. (2018)	Pilot RCT	*n* = 23 Age = 51 ± 12	GI (vs journalling) Individual 60 min, 1/week, 10 weeks	+ Fatigue (FSS) + Depression (BDI-II)	-	+ QoL (MS-QOL-54)	-	Low
Nazari et al. (2018)	RCT	*n* = 75 Age = 34 ± 6 EDSS ≤ 7.5, Female, Chronic pain	PMR+BRT (vs (1) reflexology, (2) TAU) Unclear 40min, 2/week, 4 weeks	+ Pain (NRS)	-	-	-	Low
Novais et al. (2016)	RCT	*n* = 40 Age = 30 ± 12	PMR (vs no intervention) Self-practice Unclear, 1/day, 8 weeks	+ Stress (PSS) Respiration (RR) Cardiovascular functioning (HR, BP)	-	-	-	Low
Safi (2015)	RCT	*n* = 30 Age = 28 ± 8 Female, Major Depressive Disorder	PMR (vs TAU) Group 60 min, 2/week, 6 weeks	+ Depression (BDI-II)	-	-	-	Low
Mackay et al. (2015)	RCT	*n* = 40 Age = 46 ± 12	PMR+DB+RR-and EMG-biofeedback (vs PMR+DB) Group & self-practice 60 min, 1/week & 2/day, 3 weeks	Muscle tension (EMG trapezius) Respiration (RR) + Fatigue (FSS) Depression, + Anxiety & + Stress (DASS)	-	Coping (CISS)	-	Moderate
Masoudi et al. (2013)	RCT	*n* = 70 Age = 30 ± 6	PMR (vs single session relaxation) Group & self-practice Unclear, 5 sessions & 1/day, 12 weeks	+ Pain (VAS, Verbal rating scale, MPQ)	-		-	Low
Moriya, Ikeda (2013)	Case series	*n* = 4 Age = 30–49 Fatigue	PMR Self-practice & telehealth 28 min, 1/day, 12 weeks	Fatigue (VAS) Mood (POMS)	-	QoL (SF-38)	Experiences (diary)	Low
Dayapoğlu, Tan (2012)	Quasi-experimental	*n* = 35 Age = 38 ± 10 EDSS ≤ 5, FSS ≥ 4	PMR Group & self-practice Unclear, 1 session & 1/day, 6 weeks	+ Fatigue (FSS) + Sleep (PSQI)	-	-	-	Moderate
Artemiadis et al. (2012)	RCT	*n* = 73 Age = 40 ± 11 EDSS < 4	PMR (vs waiting list) Self-practice 25 min, 2/day, 8 weeks	Anxiety (STAI) + Depression (BDI) + Stress (PSS)	-	Health locus of control (HLC scale)	Compliance (self-report checklist) + Symptom frequency and intensity (diary)	Moderate
Ghafari et al. (2009)	Quasi-experimental	*n* = 66 Age = 32 ± 7	PMR (vs no intervention) Group & self-practice 20–30 min, 1 session & daily, 8 weeks	-	-	+ QoL (SF-8 Health Survey)	Performance (self-reported checklist)	Moderate
Mackereth et al. (2009)	RCT, cross-over	*n* = 53 Age = 50 ± 11	PMR (vs reflexology) Group 40 min, 1/week, 6 weeks	+ Anxiety (SAI) + Cardiovascular Functioning (BP, HR) Stress biomarker (Salivary cortisol level) + General health (SF-36, GHQ-28	-	-	-	Low
Sutherland et al. (2005)	Pilot RCT	*n* = 26 Age = 42 ± 8.07	AT (vs no intervention) Group & self-practice Unclear, 1/week & 1/day, 10 weeks	Mood (POMS-SF) + Depression (CES-D)	-	Quality of life (MSQOL-53) Social support (MSPSS)	-	Low
Rohrmann et al. (2001)	Quasi-experimental	*n* = 60 Age = 46 ± 11 MS, Back pain, Healthy	AT (vs PMR) Group 12 min, Unclear, 3 days	+ Immune biomarker (salivary Immunoglobulin A level) + Relaxation (NRS)	-	Disease processing (FKV)	Perception, Satisfaction, Performance (self-report)	Moderate
**Stroke**								
Park et al. (2025)	Case series	*n* = 3 Age = 51 Sleep disorder	RR-biofeedback Individual 30 min, 3 sessions, 9-15 weeks	Sleep (RCSQ, PSQI,ISI)	-	-	-	Moderate
Mohd Nordin et al. (2025)	RCT	*n* = 66 Age = 48 ± 3	AT (vs physiotherapy) Individual & self-practice 20 min, 3/week, 12 weeks	+ Anxiety (HADS-A) + Depression (HADS-D)	+ ADLs (MBI)	+ Recovery Locus of Control (RLOC)	-	Moderate
El-Helou, et al. (2024)	Case series	*n* = 12 Age = 61 ± 15 Aphasia	Kalmer (PMR+GI+DB) Self-practice & telehealth 10 min, 5/week, 5 weeks	Anxiety (BOA) Depression (SADQ-II)	Communication (carer-coast)	-	Feasibility (recruitment, retention) Adherence (video analytics)	Moderate
Wang et al. (2024)	Quasi-experimental	*n* = 62 Age = 60 ± 12 Ischaemic, ≤ 3 months	HRV-biofeedback (vs TAU) Individual & self-practice (portable device) 8x20 min, 1/week & 1/day, 12 weeks	+ Cardiovascular Functioning (HR) + HRV + Cognition (MOCA) + Sensorimotor impairment (UE-FMA) Stress (PSS) + Anxiety (HADS-A) Depression (HADS-D)	-	-	-	Moderate
El-Helou et al. (2023)	Qualitative co-design	*n* = 12 Age = 61 ± 14 Aphasia	Kalmer (PMR+GI+DB) Focus groups	-	-	-	Needs, Preferences, Acceptability (focus groups)	High
Wang et al. (2022)	Mixed-method	*n* = 9 Age = 52 ± 6 Aphasia	GI+AT+breath awareness Self-practice 15 min, 1/day, 5 weeks	-	-	-	Feasibility (Feasibility scale, focus group) Compliance (diary)	Moderate
Chang et al. (2020)	RCT	*n* = 40 Age = 67 ± 10 Stroke < 1 week	HRV-biofeedback (vs TAU) Individual & self-practice (DB) 2x10 min, 4 sessions & 2/day, 12 weeks	+ HRV + Cardiovascular Functioning (HR) + Cognition (MMSE) + Anxiety (HADS-A) + Depression (HADS-D)	-	-	-	Moderate
Wang et al. (2019)	Mixed-method	(1) *n* = 13 Age = 51–76 (2) *n* = 38, n = 18 (focus groups) Age = 51–76	(1) GI+AT+counting+word repetition+breath awareness+movement (2) GI+AT+breath awareness Focus groups & Interviews	Anxiety (HADS-A)	ADLs (BI)	Self-efficacy (DLSES)	(2) Feasibility (recruitment, retention) Preferences, Acceptability (interviews, focus groups, questionnaire)	High
De Luca et al. (2018)	Case study	*n* = 1 Age = 58 Severe anxiety & Mild intellectual disability	DB (VR vs no VR) Individual 40 min, 3/week, 4 weeks	Cognition (MOCA) Respiration (oxygen level) Cardiovascular parameter (HR) Attention (Attention Matrices Test) Depression (HRS-D) Anxiety (HRS-A)	ADLs (FIM)	Coping (Cope- NVI)	-	Moderate
Golding et al. (2016, 2017, 2018)	Pilot RCT	*n* = 21 Age = 65 ± 8 HADS-A > 6	AT (vs waiting list) Self-practice 20 min, 5/week, 4 weeks	+ Anxiety (HADS-A) Depression (HADS-D)	-	-	Feasibility (recruitment, retention, practicability) Experiences, Adherence (diary)	Moderate
Kneebone et al. (2014)	Quasi-experimental	*n* = 55 Age = 74 ± 14	AT Individual 15–30 min, 1 session, 1 year	+ Muscle tension (TRCs)	-	-	-	Moderate
**SCI**								
Monden et al. (2024)	Pilot RCT	*n* = 30 Age = 42 ± 13 Tetraplegia, Anxiety	HRV-biofeedback (vs sham-biofeedback) Self-practice with remote guidance 20 min, 2/week, 4 weeks	HRV Stress (DASS-21, SUDS) Depression (DASS-21) Anxiety (DASS-21, STAI-Y)	-	-	Feasibility (recruitment, retention) Safety (adverse events)	Moderate
Schoffl et al. (2023)	Case Study	*n* = 2 Age = 54 and 58 > 11 months post-injury	HRV-biofeedback Individual, telehealth & self-practice 120–180 min, 15–30 min & 20 min, 1/week, 4/week & daily, 10 weeks	HRV Cardiovascular Functioning (ECG, BP, BPV, BRS) Anxiety (GAD-7, diary) Depression (PHQ-9) Fatigue (FSS, diary) Pain (diary) Mood (diary) Sleep quality (diary)	Disability & Participation (ADAPSS-SF, WHODAS 2.0)	Quality of life (EQ-VAS) Self-efficacy (MSES)	Safety (adverse events) Process (clinician’s report of progress and influential factors)	Moderate
Wood et al. (2021)	Pilot quasi-experimental	*n* = 20 Age = 54 ± 18	DB+GI+PMR+music Individual & self-practice 20 min, 2 sessions, 3 days	+ Pain (VAS) + Anxiety (VAS, GAD-7) Stress (PSS-10) + Relaxation (VAS)	-	-	Satisfaction (survey)	Low
Grzesiak (1977)	Case series	*n* = 4 Age = 27 ± 6 Chronic Pain	PMR+GI Individual & self-practice Unclear, 3–5 sessions, 2–4 weeks	Pain (subjective report)	Programme attendance (staff observation)	-	Benefits (subjective, staff)	Low
**CP**								
Ostojic et al. (2022)	Pilot mixed-method	*n* = 10 Age = 13 ± 6 Chronic pain	HR-biofeedback Self-practice (app) 10 min, 1/day, 4 weeks	Pain (NRS & FPS-R, Body chart & PROMIS-PPI scale) Anxiety (NRS & STAI)	-	-	-	Moderate
Mauersberger et al. (2000)	Case series	*n* = 4 Age = 12 ± 4 Mild intellectual disability	Visualization Individual 20–30 min, 5/week, 12 weeks	Muscle tension (EMG)	-	-	-	Low
Ortega (1978)	Case series	*n* = 4 Age = 29–49 Spasticity	PMR Self-practice Unclear, 2/week–1/month, 12 months	-	Manual dexterity (MRTM)	-	-	Low
**PD**								
Schlesinger et al. (2014)	Pilot quasi-experimental	*n* = 21 Age = 61 ± 12 Hoehn & Yahr stage 2–4	GI (vs music) Individual & self-practice 30 min, 2 sessions & 1/day, 12 weeks	+ Motor functioning (UPDRS Part III) + % On-time (diary)	Difficulties of daily living (PDQ-39)	-	Preference (frequency and reasons)	Moderate
**Mix**								
Hampson et al. (2020)	Quasi-experimental, cross-over	*n* = 54 Age = 15–81 Neurological condition	Mindfulness+DB+GI+PMR Individual & self-practice Unclear, 1/week, 4–12 weeks	+ Anxiety (HADS-A) + Depression (HADS-D)	-	-	Perceived changes (IACQ)	High

Studies are organized by neurological condition and year of publication. Quality ratings are not directly comparable across study designs.

MS: multiple sclerosis, SCI: spinal cord injury, CP: cerebral palsy, PD: Parkinson’s disease, RCT: randomized controlled trial in parallel design if not further specified, *n*: number of participants after allocation, age in years±1 standard deviation (SD), rounded to the nearest tenth, +: study reported statistically significant effects in favour of relaxation intervention (*p*<0.05).

*Interventions:* AT: Autogenic Training, BRT: Benson’s Relaxation Technique, DB: diaphragmatic breathing, EMG: electromyography, GI: guided imagery, HR: heart rate, HRV: heart rate variability, PMR: progressive muscle relaxation, RR: respiratory rate, TAU: treatment as usual, VR: virtual reality.

*Outcomes:* ADLs: activities of daily living, QoL: quality of life.

*Assessments:* ADAPSS-SF: Appraisals of Disability Primary and Secondary Scale – Short Form, BI: Barthel Index, BDI: Beck Depression Inventory, BFI: Brief Fatigue Inventory, BP: blood pressure, BPV: blood pressure variability, BRS: baroreflex sensitivity, CES-D: Center for Epidemiologic Studies Depression Scale, CISS: Coping Inventory for Stressful Situations, Cope-NVI: Coping Orientation to Problems Experienced – New Italian Version, DASS: Depression Anxiety Stress Scales, DLSES: Daily Living Self-Efficacy Scale, ECG: electrocardiogram, EMG: electromyography, EQ-VAS: EuroQol Visual Analogue Scale, FIM: Functional Independence Measure, FKV: Freiburg Questionnaire on Illness Processing (Freiburger Fragebogen zur Krankheitsverarbeitung), FPS-R: Faces Pain Scale – Revised, GHQ: General Health Questionnaire, GAD: Generalized Anxiety Disorder, HADS-A: Hospital Anxiety and Depression Scale – Anxiety/Depression subscale, HLC: health locus of control, HR: heart rRate, HRS-A/D: Hamilton Rating Scale for Anxiety/Depression, IACQ: Individual Assessment of Change Questionnaire, ISI: Insomnia Severity Index, MBI: Modified Barthel Index, MMSE: Mini-Mental State Examination, MOCA: Montreal Cognitive Assessment, MPQ: McGill Pain Questionnaire, MRTM: Minnesota Rate of Manipulation Test, MS-QOL: Multiple Sclerosis Quality of Life, MSPSS: Multidimensional Scale of Perceived Social Support, NRS: Numeric Rating Scale, PDQ: Parkinson’s Disease Questionnaire, PHQ: Patient Health Questionnaire, POMS: Profile of Mood States, POMS-SF: Profile of Mood States – Short Form, PROMIS-PPI: Patient Reporting Outcomes Measurement Information System Pediatric Pain Interference Scale, PSQI: Pittsburgh Sleep Quality Index, PSS: Perceived Stress Scale, RCSQ: Richards-Campbell Sleep Questionnaire, RLOC: Recovery Locus of Control, RR: respiratory rate, RSS-MS: Reece Stigma Scale for Multiple Sclerosis, SAI: State Anxiety Inventory, SADQ: Stroke Aphasic Depression Questionnaire, STAI: State-Trait Anxiety Inventory, SUDS: Subjective Units of Distress Scale, TRCs: Tension Rating Circles, TSK: Tampa Scale for Kinesiophobia, UE-FMA: Upper Extremity Fugl-Meyer Assessment, UPDRS Part III: Unified Parkinson’s Disease Rating Scale – Motor Examination, WHODAS: World Health Organization Disability Assessment Schedule.

**Fig. 2:**
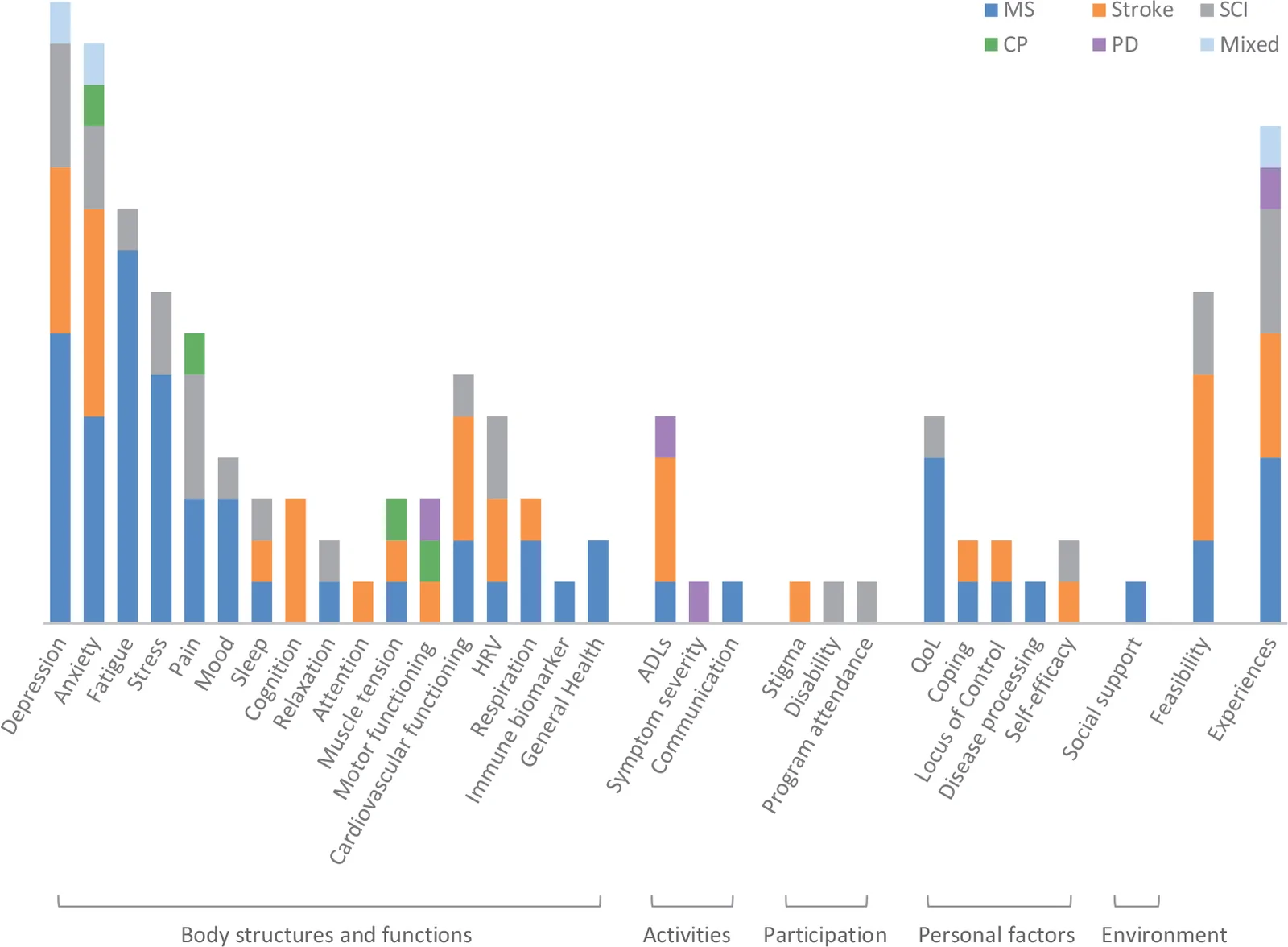
Distribution of reported study outcomes across International Classification of Functioning, Disability and Health (ICF) categories by participant population. MS: multiple sclerosis, SCI: spinal cord injury; CP: cerebral palsy; PD: Parkinson’s disease; HRV: heart rate variability; ADLs: activities of daily living; QoL: quality of life.

### General intervention characteristics

Forty-one studies investigated relaxation interventions as experimental condition. Combined interventions were most common (*n* = 11) (64, 65, 75, 83, 91, 92, 94, 99, 101, 103, 109), followed by PMR (*n* = 10) (59, 68–70, 73, 74, 89, 90, 110, 102), biofeedback (*n* = 6) (54, 55, 66, 95, 97, 98), autogenic training (*n* = 5) (67, 80, 87, 105, 107), GI (*n* = 4) (56, 58, 96, 111), Benson relaxation technique (*n* = 3) (60, 62, 63), and DB (*n* = 1) (77). One study compared PMR with autogenic training (85), and a three-arm RCT compared PMR with Benson relaxation technique and with education (57). The implementation of combined interventions varied. Some studies applied components consecutively, for example PMR before Benson relaxation technique (64, 65) or GI (109), others integrated them, for example music-assisted relaxation with mixed cues of DB, GI, and PMR (94) or PMR during biofeedback (75, 103). Four studies allowed participants to choose interventions to explore preferences and adaptation strategies (91, 92, 99, 101). Varieties of DB, autogenic training, and GI were developed in mixed-method design and subsequently tested for feasibility in stroke survivors with and without aphasia (91, 92). Similarly, the Kalmer relaxation intervention, a combination of DB, PMR, and GI, was co-designed and evaluated for home practice feasibility in stroke survivors (99, 101).

PMR was predominantly delivered as self-practice, preceded by instructional sessions and supported with auditory materials (59, 69, 73, 74, 102). One study provided additional weekly phone calls (74), while others provided neither material (57) nor prior in-person education (70, 89). Practice frequency ranged from twice daily (89) to five times weekly (110), with intervention durations of four (110) to 12 weeks (57, 69, 70). Sessions lasted 20–30 min (57, 70, 74, 89), though 5 studies did not report duration (59, 73, 110, 69, 102).

Biofeedback interventions were based on visualization of heart rate variability (54, 55, 66, 97), heart rate (98), or respiratory rate (66), and predominantly delivered in individual sessions (54, 55, 66, 97). Two studies provided portable devices for additional home practice (54, 97), and one encouraged self-practice of deep breathing (55). Two pilot studies tested home-based biofeedback with remote guidance (95) or caregiver support (98). Practice frequency ranged from once (55, 98) to five times weekly (54, 97), with intervention durations from nine days (66) to 12 weeks (54). Session length was 10 (98) to 30 min (66).

Autogenic training was delivered in group sessions (85, 105, 107), individual sessions with self-practice (67), or self-practice with auditory materials (80). Practice frequency ranged from once (105, 107) to five times weekly (80), with intervention durations from a single session (107) to 12 weeks (67). Session length was 12 (85) to 30 min (107), though one study did not report duration (105).

GI interventions were predominantly delivered individually and tailored to specific populations. For individuals with MS, a customized approach involved visualizing light entering the crown of the head and dispersing into specific colours to address physical and psychological concerns (96). Another approach involved audio recordings of relaxing scenarios with affirmations to reduce fatigue and stigma, and enhance mood (56). For individuals with Parkinson’s disease, the tailored GI script guided participants into relaxation to promote well-being and alleviate symptoms such as rigidity, tremor, and “off” episodes (58). In a case series involving children with cerebral palsy, personalized sessions were provided with gradually reduced guidance, transitioning GI into a form of self-hypnosis (111). Practice frequency ranged from once (96) to six times weekly (56), with intervention durations of 10 weeks (96), 12 weeks (56, 58), and up to one year (111). Session length was 25 (56) to 60 min (96).

### Overview per population

*Multiple sclerosis*. A total of 33 studies involving individuals with MS were included (56, 57, 59–65, 68–76, 81, 84–87, 89, 90, 93, 96, 102–106, 112). In 21, relaxation was the primary intervention under investigation. Of these, most employed RCT designs (*n* = 15) (56, 57, 62–65, 68, 69, 75, 89, 90, 102, 103), including two pilot RCTs (96, 105). Additional designs included five quasi-experimental studies (59, 60, 73, 74, 85) and one case series (70). Two studies included follow-up assessments after two and three months, respectively (65, 103).

PMR was the most frequently applied intervention (*n* = 9) (59, 68–70, 73, 74, 89, 90, 102), followed by Benson relaxation technique (*n* = 3) (60, 62, 63) and GI (*n* = 2) (56, 96). In addition, two studies employed a combined intervention of PMR and Benson relaxation technique (64, 65), and one study combined PMR and DB to investigate the additional effect of biofeedback (103). Three studies directly compared the effects of different relaxation interventions. In a three-arm RCT, PMR was compared with Benson relaxation technique and with education (57), while another study compared PMR with autogenic training (85). Further, one study investigated heart rate and respiratory rate biofeedback with PMR vs with DB (75). Other control conditions included treatment as usual, waiting list, reflexology, and journalling.

Across studies, most outcomes were related to the ICF domain body functions and structures. Fatigue was the most frequently assessed outcome (*n* = 9) (56, 57, 59, 63, 70, 73, 75, 96, 103), followed by depression (*n* = 7) (60, 64, 68, 89, 96, 103, 105). Stress was assessed both subjectively (60, 64, 89, 102, 103) and through stress-related physiological parameters such as heart rate, blood pressure, breathing rate, and salivary cortisol levels (75, 90, 102, 103). Overall, self-report questionnaires were the predominant assessment method. One study explored patient perceptions qualitatively through diary entries (70). Quality appraisal indicated that most studies were of low to moderate methodological quality, with common limitations related to blinding procedures, intervention administration and participant retention.

*Stroke*. A total of 19 studies involving stroke survivors were included (54, 55, 66, 67, 77–79, 80, 82, 88, 91, 92, 99, 101, 107, 113–116). In two cases, different outcomes from the same primary dataset were reported in separate publications (80, 113, 114 and 78, 115, 116). Consequently, the 19 studies correspond to 15 primary datasets. For clarity, references in the section refer to the first publication associated with each dataset. Relaxation was the primary intervention under investigation in 11 of the included studies (54, 55, 66, 77, 80, 91, 92, 99, 101, 107, 112). Among these, two studies each employed an RCT design (55, 80), quasi-experimental design (54, 107), and mixed-method design (91, 92) while one was a qualitative co-design study (101) followed by a feasibility case series (99). Further, another case series (66) and one case report (77) were included. One study followed up on participants after 12 months (113).

In four of the included studies, relaxation interventions were adapted to the needs of stroke survivors and consists of combinations, such as the co-designed Kalmer intervention (99, 101) or a mix of GI, autogenic training, and breath awareness (91, 92). Additionally, effects of autogenic training (*n* = 3) (80, 107, 112), or heart rate variability (*n* = 2) and respiratory rate biofeedback (*n* = 1) (54, 55, 66) were investigated. Comparators were treatment as usual, waiting list control, and physiotherapy. One single case study investigated the effects of DB in a usual versus virtual rehabilitative environment (77), while no study examined effects of PMR.

The majority of outcomes were related to the ICF domain body functions and structures, most commonly anxiety (*n* = 7) (54, 55, 67, 77, 80, 92, 99) and depression (*n* = 6) (54, 55, 67, 77, 99, 114). Perceived stress was assessed in one study (54) and physiological parameters related to stress in three (54, 55, 77). Overall, self-report questionnaires were the predominant assessment method. Additionally, stroke survivors were the only population included in this review for which qualitative data were collected and reported repeatedly (80, 91, 92, 101). Quality appraisal indicated that most studies were of moderate methodological quality, with common limitations related to blinding procedures, sampling, and intervention administration.

*Spinal cord injury.* A total of six studies involving individuals with spinal cord injury were included (94, 95, 97, 100, 108, 109). In four of these, relaxation was the primary intervention under investigation (94, 95, 97, 109). A recent pilot RCT examined the feasibility and preliminary effects of a heart rate variability biofeedback intervention on depression, anxiety, and perceived and physiological stress in individuals with tetraplegia as compared with physiological monitoring alone (95). Additionally, a neuropsychophysiological laboratory framework for heart rate variability biofeedback interventions for individuals with spinal cord injury was proposed. In an enclosed case series, feasibility and potential effects on anxiety, depression, fatigue, mood, pain, sleep quality, physiological parameters, disability levels, and quality of life were assessed (97). Another pilot quasi-experimental study investigated a combined intervention of DB, GI, and PMR practice for its feasibility and potential effects on pain, anxiety, stress, and subjective level of relaxation (94). Lastly, a case study described the effects of a combined PMR and GI intervention on pain and programme attendance in four individuals with injury-related pain (109). Across studies, self-report questionnaires were the predominant assessment method, and quality appraisal indicated that studies were of low to moderate methodological quality.

*Cerebral palsy*. Three of the included studies were conducted with individuals with cerebral palsy (98, 110, 111). In all of these, relaxation was the primary intervention under investigation. A pilot mixed-methods trial examined the acceptability and feasibility of heart rate biofeedback for chronic pain management in children with cerebral palsy (98). In a case series, the effects of PMR on manual dexterity in adults with spastic cerebral palsy was explored (110). Another case series investigated whether children with spastic quadriplegic cerebral palsy and mild intellectual disability could use hypnotic visualization to reduce muscle tone (111). The quality appraisal indicated low methodological quality for the case series and moderate quality for the mixed-method trial.

*Parkinson’s disease*. One pilot quasi-experimental study involving individuals with Parkinson’s disease was included. Participants received a single session of GI and a single session of relaxing music. The study assessed intervention preferences, motor functioning, and difficulties in daily living (58). Methodological quality was rated as moderate.

*Mixed population*. One quasi-experimental trial included individuals with long-term neurological conditions, such as stroke, MS, Parkinson’s disease, acquired brain injury, spinal neuropathy, and brain tumour. The study investigated the effects of a combined intervention of DB, GI, PMR, and mindfulness practice on depression and anxiety (83). Methodological quality was rated as high.

## DISCUSSION

Research on relaxation interventions in neurological rehabilitation spans multiple regions and decades. The evidence base comprises 63 sources with quantitative and qualitative designs. Across these, relaxation interventions were consistently reported as feasible and well accepted, and associated with improved psychological well-being. However, the evidence is heterogeneous, based on small samples, generally of low to moderate quality, and lacks pooled analyses. While findings highlight the potential of relaxation interventions in neurological rehabilitation, they also expose substantial knowledge gaps requiring further research.

### Evidence across populations

The number of studies is unevenly distributed across neurological populations. The comparatively strong representation of MS in the current review adds to the existing evidence base for MS. Kneebone et al. systematically reviewed 28 studies on relaxation and related therapies in people with MS and reported beneficial effects on depression, anxiety, stress, and fatigue, although the evidence was limited by substantial risk of bias and heterogeneity too (40). Our review extends the MS-specific synthesis by mapping the literature on relaxation interventions in neurological rehabilitation more broadly. However, the majority of included studies remain concentrated in MS and stroke populations, with many other neurological conditions being underrepresented. This could be due to several reasons. First, research activity tends to cluster around prevalent conditions, where larger patient cohorts, established clinical networks, and stronger funding streams can facilitate study design and recruitment (117). Further, the widespread use of complementary interventions among people with MS may drive research interest (118). Across other conditions, participation in structured self-managed programmes may be limited by motor and cognitive impairments, and conventional neurological settings often remain centred on physical recovery (119–121). Despite evidence that stress affects motor function (17, 122–124), the findings of this review suggest that relaxation interventions are not yet primarily associated with functional recovery. Finally, the eligibility criteria led to the exclusion of some conditions, like migraine or headache, despite substantial research activity (125, 126). The uneven distribution limits generalizability and risks overlooking population-specific needs.

### Evidence across interventions

The wide variability in intervention characteristics reflects the importance of population-specific adaptations. In individuals with MS, PMR was the most frequently studied intervention. In studies involving stroke survivors, it was not evaluated as an experimental condition, whereas cognitively and mentally guided interventions, such as autogenic training or biofeedback, were frequently applied. Further, inclusion criteria in these trials were more restrictive than in studies with other populations, enabling the investigation of more homogeneous subgroups and facilitating qualitative exploration and participatory design of relaxation interventions. Findings from these studies highlight substantial inter-individual variability alongside general preference patterns (92, 101). In parallel, direct head-to-head comparison of relaxation interventions was rare and did not provide evidence of objective superiority. Across the three studies identified, multiple interventions showed beneficial effects (75, 103, 85). Findings of similar studies in non-neurological samples also suggest functional equivalence for psychological outcomes, despite modest differences in physiological patterns (23, 127). Taken together, the observed heterogeneity of interventions, qualitative insights, and limited comparative evidence neither establishes equivalence between interventions nor supports conclusions concerning the superiority of specific interventions for particular populations. Thus, no clear hierarchy of relaxation interventions can currently be derived from the available evidence.

### Evidence across outcomes

Most included studies reported outcomes within the ICF body structure and function domain, with an emphasis on psychological functioning. Although a wide range of outcomes were assessed overall, measures related to activities, participation, and contextual factors were rather rare. This is consistent with broader patterns in stress-management research in neurological rehabilitation. Scoping reviews on mindfulness‑based interventions and psychological stress‑management strategies in stroke rehabilitation similarly reported limited to no evaluation of activity‑ and participation‑related outcomes (39, 38). This gap is critical, given that rehabilitation primarily aims to optimize functioning and participation, as emphasized by the World Health Organization (128). Crucially, patients also find activity- and participation-related outcomes highly relevant (129). In the few studies that did examine outcomes beyond the body function and structure domain, findings were inconsistent, though some studies suggest potential benefits for activities of daily living and quality of life (74, 96, 59, 112). Overall, the narrow outcome focus may underestimate the broader therapeutic potential of relaxation interventions in the context of neurological rehabilitation. Comprehensive outcome frameworks are needed that capture effects across multiple ICF domains, especially on activity and participation level.

A second shortcoming relates to the measurement of outcomes. Although only interventions explicitly designed to induce a physiological relaxation response were eligible, only a minority of studies actually measured physiological markers related to relaxation or autonomic arousal. Similarly, only a quarter of all included studies measured perceived stress or relaxation. This limits comparability and raises concerns regarding construct validity, as intervention effects cannot be meaningfully interpreted without confirming successful induction of relaxation. Therefore, purposeful selection of stress and relaxation measures is essential (130). Importantly, physiological and perceived stress are related but not interchangeable (131, 132). To enhance validity and comprehensiveness, future trials should adopt a multidimensional approach of measuring relaxation and stress (130, 133).

### Quality assessment

The methodological quality of the included studies was predominantly low to moderate, limiting confidence in effect estimates. Common methodological weaknesses included inadequate allocation concealment and inconsistent blinding of outcome assessors, increasing the risk of selection and detection bias. Non-equivalent control conditions and incomplete follow-up without intention-to-treat analysis further raised concerns regarding performance and attrition bias. Reporting on intervention delivery was often limited, with missing information on administrator training and measurement reliability. No study reported adverse effects or negative outcomes, which may reflect intervention safety but also raises the possibility of selective reporting or publication bias. Collectively, these methodological limitations should be considered when interpreting the current evidence base.

### Strengths and limitations

This scoping review offers the first comprehensive overview of the current state of research on relaxation interventions in neurological rehabilitation. By systematically mapping the broad and heterogeneous literature, it establishes a foundational reference point for researchers and clinicians. The transparent methodology, structured synthesis of qualitative and quantitative literature, ICF-based categorization of outcomes, and critical appraisal of included studies enhance the accessibility and interpretability of the literature and support the identification of research priorities. Nevertheless, several limitations should be acknowledged. First, the evidence synthesis was intentionally broad rather than in-depth. While this approach enabled a comprehensive mapping of the literature, it did not allow for detailed analysis or definitive conclusions. Second, although grey literature was included, publication bias cannot be excluded, and some relevant studies may have been missed. Study selection was further influenced by predefined eligibility criteria and challenges in delineating conceptual boundaries between relaxation, mindfulness, and related stress‑management approaches, particularly in studies evaluating combined interventions or biofeedback and neurofeedback protocols with indirect or abstract outcomes. Third, although measures were taken to reduce bias, some subjectivity remains. While screening and critical appraisal were conducted independently by two researchers, data extraction was performed by a single reviewer due to resource constraints. The data charting form was piloted to refine extraction items and terminology, but no independent second-reviewer verification of the extracted data was performed. Together, these limitations define boundaries for interpretation and underscore the need for more methodologically robust and conceptually aligned research approaches. Despite these limitations, the review identified numerous avenues for future research.

### Recommendations for future research

The current evidence base for relaxation interventions in neurological rehabilitation is limited, highlighting the need for a more structured and methodologically robust research agenda. First, evidence needs to spread across populations with diverse neurological conditions to improve generalizability and avoid overlooking population‑specific needs. Second, studies should investigate how a person-centred approach to relaxation can be systematically described and evaluated. Transparent reporting of intervention adaptations and decision‑making processes should be standard to enable transferability across settings while preserving the responsiveness to individual rehabilitation needs. Qualitative or co-design strategies are needed to explore the populations’ perspective and head‑to‑head trials to evaluate comparative effectiveness. Third, outcome frameworks should capture a broader spectrum of effects, including activity and participation measures. Validity must be ensured by incorporating both perceived relaxation and physiological stress markers. Lastly, future research must move toward high-quality study designs and robust methodological rigour. This includes adequately powered sample sizes, transparent randomization and allocation procedures, blinding of outcome assessors, equivalent control conditions, complete reporting of follow-up, and intention-to-treat analyses. Collectively, these recommendations aim to guide future research towards generating robust evidence on the role and effectiveness of relaxation interventions in neurological rehabilitation.

### Clinical implications

For clinicians, the findings suggest that relaxation interventions are feasible and may be considered as supportive components of neurological rehabilitation, particularly when addressing stress, psychological well-being, or patient self-management. However, given the heterogeneity of interventions, populations, and outcome measures, the current evidence does not support recommending one specific relaxation intervention over another or drawing firm condition-specific conclusions as to effectiveness. Therefore, functional and cognitive abilities, preferences, rehabilitation goals, and the resources available can guide intervention selection at this stage. This person-centred approach may enhance engagement, adherence, and therapeutic outcomes (92, 134–136), but should be understood as a pragmatic clinical implication rather than evidence of equivalence between interventions. Where relaxation interventions are used, clinicians should consider monitoring perceived stress and relaxation levels alongside broader rehabilitation outcomes such as activities, participation, and quality of life.

In conclusion, this scoping review provides a structured overview of the literature on relaxation interventions in neurological rehabilitation by mapping populations, intervention characteristics, and outcome domains. The findings reveal a heterogeneous evidence landscape, within which several patterns emerge. Relaxation interventions have primarily been studied in people with MS and stroke, are generally reported as feasible and well accepted, and were often adapted to specific functional needs and preferences. Reported outcomes most commonly relate to psychological functioning. Key gaps in the evidence base include limited research into less prevalent conditions, few qualitative designs and comparative effectiveness studies, as well as limited assessment outcomes relating to activities, participation, and perceived and physiological stress. Overall, this review provides a comprehensive overview of the application of relaxation interventions in the context of neurological rehabilitation research. It clarifies the key patterns and gaps that need to be considered when interpreting current findings and shaping subsequent research in this field.

## Supplementary Material


