# Supplementary material for: RELAXATION INTERVENTIONS IN NEUROLOGICAL REHABILITATION: A SCOPING REVIEW
Source: J Rehabil Med. 2026 Oct 8;58:45972. doi: 10.2340/jrm.v58.45972 (PMC13656073; doi:10.2340/jrm.v58.45972)
Supplement: Supplementary file 1 [file JRM-58-45972-s1.pdf]

SUPPLEMENTRY TABLE LEGENDS*

Table SI: Eligibility criteria

Table SII: Keywords and MeSH-Terms of the literature search

Table SIII: Characteristics of included studies with relaxation as control condition

Table SIV: Detailed critical appraisal results

Appendix S1

Table SI: Eligibility criteria

	Inclusion	Exclusion
**Population**	Individuals who have received a formal diagnosis of a neurological condition (classified by ICD-10)	Individuals with a diagnosis other than a neurological condition
	Any age, gender and nationality	Mixed population, if results not reported separately or if less than 80% of the sample has not received a formal diagnosis of a neurological condition
**Concept**	Interventions explicitly aimed at inducing a physiological relaxation response (e.g. autogenic training, guided/visual imagery, progressive muscle relaxation, deep breathing exercises, biofeedback-interventions based on heart rate variability, heart rate, breathing pattern, skin conductance, hand temperature or brain activity)	Interventions that do not primarily aim to induce a physiological relaxation response such as psychotherapeutic interventions (e.g. talk therapy, neurolinguistics psychology) or respiratory muscle training, motor imagery interventions
	Studies must link intervention to a state of relaxation	Mind-body interventions that primarily focus on other concepts such as mindfulness (e.g. meditation, MBSR, body scan, basic body awareness therapy) or movement (yoga, qigong, tai-chi, Alexander technique, Feldenkrais method) or expressive therapies (art, music, dance, theatre)
	Any duration or intensity	

		Interventions focusing on local relaxation, e.g. EMG biofeedback-interventions based on specific (local) muscle activity, massage therapy or acupuncture/-pressure
		Relaxation interventions in conjunction with other treatment modalities, if no clear distinction was made or less than 80% focuses on relaxation
**Context**	Multimodal neurological rehabilitation	Neurological conditions that usually do not require multdisciplinary neurological rehabilitation (e.g. migraine, epilepsy, dementia)
		Relaxation interventions not suitable for physiotherapeutic settings or self-administration (e.g. acupuncture, hypnosis)
**Study Character-istics**	Original research: all primary research studies (e.g., randomized controlled trials, controlled clinical trials, cohort studies, case-control studies, case series)	Poster/abstract presentations without full-text
		Editorials, comments, biographies
	Feasibility studies/ pilot-studies	Guidelines and consensus development
	Meta-analyses/reviews	Books (chapters)
	Qualitative and quantitative methods	Inaccessible full-text
	No time frame	Language other than English, German, Dutch

Appendix S2

Table SII: Keywords and MeSH-Terms of the literature search

Population	Concept	Exclusion
Keywords	*Keywords*	*Keywords*
neurological disorder*	relaxation therap*	migraine
neurologic disorder*	relaxation techni*	headache
neurological disease*	relaxation training	electromyography*
neurologic disease*	therapeutic relaxation	
neurologic condition*	relaxation response	
neurological condition*	stress reduction	
nervous system disease*	guided relaxation	
nervous system disorder*	autogenic training	
neurological rehabilitation	progressive muscle relaxation	
neurorehabilitation	guided imagery	
stroke*	deep breathing exercise*	
cerebrovascular accident*	biofeedback*	
spinal cord disease*		
spinal cord injur*		
multiple sclerosis		
cerebral palsy		
MeSH-Terms	*MeSH-Terms*	
Nervous System Diseases	Relaxation therapy	
	Breathing exercises	
	Biofeedback, Psychology	
	autogenic training	
	Imagery, Psychotherapy	

Literature string per database

PubMed

("neurological disorder*"[Title/Abstract] OR "neurologic disorder*"[Title/Abstract] OR "neurological disease*"[Title/Abstract] OR "neurologic disease*"[Title/Abstract] OR "neurologic

condition*"[Title/Abstract] OR "neurological condition*"[Title/Abstract] OR "nervous system disease*"[Title/Abstract] OR "nervous system disorder*"[Title/Abstract] OR "neurological rehabilitation"[Title/Abstract] OR "neurorehabilitation"[Title/Abstract] OR "stroke*"[Title/Abstract] OR "cerebrovascular accident*"[Title/Abstract] OR "spinal cord disease*"[Title/Abstract] OR "spinal cord injur*"[Title/Abstract] OR "multiple sclerosis"[Title/Abstract] OR "cerebral palsy"[Title/Abstract]) OR ("Central Nervous System Diseases/rehabilitation"[Mesh] OR "Central Nervous System Diseases/therapy"[Mesh]) AND ("relaxation therap*"[Title/Abstract] OR "therapeutic relaxation"[Title/Abstract] OR "relaxation techni*"[Title/Abstract] OR "relaxation training"[Title/Abstract] OR "guided relaxation"[Title/Abstract] OR "Autogenic Training"[Title/Abstract] OR "progressive muscle relaxation"[Title/Abstract] OR "relaxation response"[Title/Abstract] OR "stress reduction"[Title/Abstract] OR "deep breathing exercise*"[Title/Abstract] OR "biofeedback*"[Title/Abstract]) OR ("Relaxation Therapy"[MeSH Terms] OR "Breathing Exercises"[MeSH Terms] OR "biofeedback, psychology"[MeSH Terms] OR "Autogenic Training"[MeSH Terms] OR "imagery, psychotherapy"[MeSH Terms]) NOT (electromyographic[Title/Abstract]) OR (headache[Title/Abstract]) OR (migraine[Title/Abstract])

FILTER:, Dutch, English, German, Humans, Adaptive Clinical Trial, Books and Documents, Case Reports, Classical Article, Clinical Study, Clinical Trial, Clinical Trial, Phase I, Clinical Trial, Phase II, Clinical Trial, Phase III, Clinical Trial, Phase IV, Collected Work, Comparative Study, Controlled Clinical Trial, Corrected and Republished Article, Equivalence Trial, Evaluation Study, Historical Article, Interview, Introductory Journal Article, Meta-Analysis, Multicenter Study, Observational Study, Pragmatic Clinical Trial, Preprint, Randomized Controlled Trial, Review, Scientific Integrity Review, Systematic Review

Embase (Ovid)

(neurological disorder* or neurologic disorder* or neurological disease* or neurologic disease* or neurologic condition* or neurological condition* or nervous system disease* or nervous system disorder* or neurological rehabilitation or neurorehabilitation or stroke* or cerebrovascular accident* or spinal cord disease* or spinal cord injur* or multiple sclerosis or cerebral palsy).ab,ti. OR central nervous system disease/rh, th [Rehabilitation, Therapy] AND (relaxation therap* or therapeutic relaxation or relaxation techni* or relaxation training or guided relaxation or autogenic training or progressive muscle relaxation or relaxation response or stress reduction or deep breathing exercise* or biofeedback*).ab,ti. OR exp relaxation training/ or muscle relaxation/ or autogenic training/ or guided imagery/ or exp *biofeedback/ NOT (electromyographic or headache or migraine).ab,ti

FILTER: Dutch, English, German, Human, article, article in press, books, chapter, data paper, preprint (unpublished, non-peer reviewed), review, short survey, tombstone

PsycInfo (Ebsco)

TI (neurological disorder* or neurologic disorder* or neurological disease* or neurologic disease* or neurologic condition* or neurological condition* or nervous system disease* or nervous system disorder* or neurological rehabilitation or neurorehabilitation or stroke* or cerebrovascular accident* or spinal cord disease* or spinal cord injur* or multiple sclerosis or cerebral palsy) OR AB (neurological disorder* or neurologic disorder* or neurological disease* or neurologic disease* or neurologic condition* or neurological condition* or nervous system disease* or nervous system disorder* or neurological rehabilitation or neurorehabilitation or stroke* or cerebrovascular accident* or spinal cord disease* or spinal cord injur* or multiple sclerosis or cerebral palsy) OR DE "Cerebrovascular Disorders" OR DE "Nervous System Disorders" AND TI (relaxation therap* or therapeutic relaxation or relaxation techni* or relaxation training or guided relaxation or autogenic training or progressive muscle relaxation or relaxation response or stress reduction or deep breathing exercise* or biofeedback*) OR AB (relaxation therap* or therapeutic relaxation or relaxation techni* or relaxation training or guided relaxation or autogenic training or progressive muscle relaxation or relaxation response or stress reduction or deep breathing exercise* or biofeedback*) OR MM "Relaxation Therapy" OR DE "Breathing Techniques" OR DE "Progressive Relaxation Therapy" OR DE "Autogenic Training" OR DE "Guided Imagery" OR DE "Muscle Relaxation" OR DE "Biofeedback Training" NOT TI (electromyographic or headache or migraine) OR AB (electromyographic or headache or migraine)
FILER: Englisch, German (no Dutch article identified)

AMED

TI (neurological disorder* or neurologic disorder* or neurological disease* or neurologic disease* or neurologic condition* or neurological condition* or nervous system disease* or nervous system disorder* or neurological rehabilitation or neurorehabilitation or stroke* or cerebrovascular accident* or spinal cord disease* or spinal cord injur* or multiple sclerosis or cerebral palsy) OR AB (neurological disorder* or neurologic disorder* or neurological disease* or neurologic disease* or neurologic condition* or neurological condition* or nervous system disease* or nervous system disorder* or neurological rehabilitation or neurorehabilitation or stroke* or cerebrovascular accident* or spinal cord disease* or spinal cord injur* or multiple sclerosis or cerebral palsy) AND TI (relaxation therap* or therapeutic relaxation or relaxation techni* or relaxation training or guided relaxation or autogenic training or progressive muscle relaxation or relaxation response or stress reduction or deep breathing exercise* or biofeedback*) OR AB (relaxation therap* or therapeutic relaxation or relaxation techni* or relaxation training or guided relaxation or autogenic training or progressive muscle relaxation or relaxation response or stress reduction or deep breathing exercise* or biofeedback*) NOT TI (electromyographic or headache or migraine) OR AB (electromyographic or headache or migraine)

FILTER: Englisch, German (no Dutch article identified)

CINAHL

TI (neurological disorder* or neurologic disorder* or neurological disease* or neurologic disease* or neurologic condition* or neurological condition* or nervous system disease* or nervous system disorder* or neurological rehabilitation or neurorehabilitation or stroke* or cerebrovascular accident* or spinal cord disease* or spinal cord injur* or multiple sclerosis or cerebral palsy) OR AB (neurological disorder* or neurologic disorder* or neurological disease* or neurologic disease* or neurologic condition* or neurological condition* or nervous system disease* or nervous system disorder* or neurological rehabilitation or neurorehabilitation or stroke* or cerebrovascular accident* or spinal cord disease* or spinal cord injur* or multiple sclerosis or cerebral palsy) OR MH "Nervous System Diseases/RH/TH" AND TI (relaxation therap* or therapeutic relaxation or relaxation techni* or relaxation training or guided relaxation or autogenic training or progressive muscle relaxation or relaxation response or stress reduction or deep breathing exercise* or biofeedback*) OR AB (relaxation therap* or therapeutic relaxation or relaxation techni* or relaxation training or guided relaxation or autogenic training or progressive muscle relaxation or relaxation response or stress reduction or deep breathing exercise* or biofeedback*) OR MH "Relaxation Techniques" OR MH "Guided Imagery" OR MH "Biofeedback" NOT TI (electromyographic or headache or migraine) OR AB (electromyographic or headache or migraine)

FILTER: Englisch, German, Dutch/Flemish

Web of Science

(TI=(neurological disorder* or neurologic disorder* or neurological disease* or neurologic disease* or neurologic condition* or neurological condition* or nervous system disease* or nervous system disorder* or neurological rehabilitation or neurorehabilitation or stroke* or cerebrovascular accident* or spinal cord disease* or spinal cord injur* or multiple sclerosis or cerebral palsy) OR AB=(neurological disorder* or neurologic disorder* or neurological disease* or neurologic disease* or neurologic condition* or neurological condition* or nervous system disease* or nervous system disorder* or neurological rehabilitation or neurorehabilitation or stroke* or cerebrovascular accident* or spinal cord disease* or spinal cord injur* or multiple sclerosis or cerebral palsy)) AND (TI=(relaxation therap* or therapeutic relaxation or relaxation techni* or relaxation training or guided relaxation or autogenic training or progressive muscle relaxation or relaxation response or stress reduction or deep breathing exercise* or biofeedback*) OR AB=(relaxation therap* or therapeutic relaxation or relaxation techni* or relaxation training or guided relaxation or autogenic training or progressive muscle relaxation or relaxation response or stress reduction or deep breathing exercise*

or biofeedback*)) NOT (TI=(electromyographic or headache or migraine) OR AB=(electromyographic or headache or migraine))
FILTER: English or German (Languages) and Article or Review Article or Proceeding Paper or Book Chapters or Early Access or Correction (Document Types) and Rehabilitation or Clinical Neurology or Medicine Research Experimental or Psychiatry or Multidisciplinary Sciences or Integrative Complementary Medicine or Psychology Clinical or Psychology or Behavioral Sciences or Health Care Sciences Services or Primary Health Care or Psychology Applied (Web of Science Categories)

Grey Literature

ClinicalTrial.gov

Condition: neurological diseases or conditions

Intervention: relaxation

FILTER: all studies with results

ProQuest Dissertation&Thesis

"neurological condition" AND relaxation

Appendix S3

Table SIII: Characteristics of included studies with relaxation as control condition

Main author & Year	Study design	Participants *n, age (SD), inclusion criteria*	Interventions *Type, Setting, Length, Frequency, Duration*	Outcomes *(Assessments)* Body functions/structure	Activities/ Participation	Environmental/ Personal factors	Feasibility/ Experiences	Quality Rating
**MS**								
Brambila-Tapia et al. (2022)	RCT	n = 15<br>age = 41 ± 9<br>EDSS ≤ 6	GI (vs Hypnoanalysis)<br>Group & self-practice<br>30–40min, 1/week & daily, 10 weeks		Disability *(RMDQ)*	QoL *(SF-36)*		Low
Ozkul et al. (2020a)	RCT	n = 39<br>age = 32 ± 12<br>EDSS < 6	PMR (vs Pilates in VR environment vs Pilates and balance exercises)<br>Self-practice<br>15–20min, 2/week, 8 weeks	Balance *(BBS, Posturography)*<br>Fatigue *(FSS)*	Walking *(TUG)*			Low
Ozkul et al. (2020b)	RCT	n = 20<br>age = 44 ± 14<br>EDSS 2 - 5.5	PMR (vs exercise)<br>Self-practice<br>15–20min, 2/week, 6 weeks	Balance *(MSOT, BBS, ABC)*<br>Cognition *(BRB-N, MSNQ)*	Walking *(TUG, FGA, MSWS-12)*<br>Manual dexterity *(NHPT)*			Moderate
Ozkul et al. (2020c)	RCT	n = 34<br>age = 36 ± 9<br>EDSS < 4	PMR (vs exercise)<br>1 educational session & self-practice<br>15–20min, 3/week, 8 weeks	Cognition *(BRB-N)*<br>Fatigue *(FIS)*<br>Depression *(BDI)*	Walking *(6-MWT)*	QoL *(MSQoL-54)*		Moderate

		cognitive<br>impairment						
Giovannetti et al. (2016)	Pilot mixed-methods	n = 37<br>age = 46 ± 9<br>CD-RISK < 83	AT (vs ACT)<br>Group<br>60min, 1/week, 7 weeks and 1 session 5 weeks after 7th session	Anxiety *(HADS-A)*<br>Depression *(HADS-D)*<br>Stress *(PSS)*<br>Mindfulness *(MAAS)*<br>Acceptance *(AAQ-II, CompACT)*<br>Resilience *(CD-RISC 25)*<br>Cognitive defusion *(DDS)*		QoL *(MSQOL-54, SEIQoL-DW)*<br>Values *(VLQ)*	Experiences *(interviews)*<br>Satisfaction *(purpose-build questionnaire)*	Moderate
Fox et al. (2016)	RCT	n = 100<br>age = 54 ± 10<br>EDSS 4 - 6.5	PMR (vs Pilates vs exercise)<br>Individual & self-practice<br>60min, 1/month & daily, weekly telephone contact, 12 weeks	Balance *(Functional reach, ABC)*	Walking *(10MWT, MSWS-12)*<br>Dual task *(NRS)*			Moderate
Carletto et al. (2017)	RCT	n = 42<br>age = 40 ± 11<br>PTSD diagnosis and symptoms *(CAPS)*<br>MS-illness considered as	DB+PMR+GI+cue-controlled and rapid relaxation (vs EMDR)<br>Individual<br>60min, 1–2/every 2 weeks (10 sessions total), 12-15 weeks	PTSD severity *(CAPS)*<br>Anxiety *(HADS-A)*<br>Depression *(HADS-D, CMDI)*<br>Fatigue *(FSS)*		QoL *(FAMS)*<br>Distress through traumatic event *(IES-R)*<br>Trauma load *(TAQ)*		Moderate

		only traumatic event *(IES-R)*						
Kos et al. (2016)	RCT	n = 31<br>age = 41 ± 9<br>EDSS ≤ 5<br>VAS fatigue ≥ 60	PMR+AT+GI (vs SMOoTh program)<br>Individual<br>60–90min, unclear, 3 weeks	Fatigue *(MFIS)*	ADLs *(COPM)*	QoL *(SF-36)*		High
Castro-Sánchez et al. (2012)	RCT	n = 73<br>age = 48 ± 11<br>EDSS ≤ 7.5, VAS pain score > 4 for at least 2 months	PMR + DB (vs Ai Chi)<br>Group<br>60min, 2/week, 20 weeks	Pain *(VAS, MPQ)*<br>Spasm *(VAS)*<br>Fatigue *(MFIS, FFS)*<br>Depression *(BDI-II)*	ADLs *(BI)*			Moderate
Nordin and Rorsman (2012)	RCT	n = 21<br>age = 46 ± 10<br>EDSS < 6, BDI ≥ 10 and/or HADS > 8	AR (vs ACT)<br>Group & self-practice<br>Unclear, 5 sessions in total & home-practice, 15 weeks	Anxiety *(HADS-A)*<br>Depression *(HADS-D)*<br>Acceptance *(AAQ-II)*				Moderate
Jensen et al. (2009)	Quasi-experi-mental trial	n = 22<br>age = 52 ± 12<br>pain daily NRS ≥ 4	PMR (vs self-hypnosis)<br>Individual & self-practice<br>Unclear, up to 10 sessions, unclear	Pain *(NRS, interview)*	Pain interference *(modified PIS from BPI)*	Hypnotizability *(SHCS)*	Adherence (*interview)*<br>Treatment Expectancy *(TES)*<br>Effects *(interview)*	Moderate

van Kessel et al. (2008)	RCT	n = 72<br>age = 45 ± 10<br>EDSS ≤ 6<br>FSS ≥ 4	DB+PMR+GI+cue-controlled and rapid relaxation (vs CBT)<br>Individual & self-practice<br>50min, 1/week, 8 weeks	Fatigue *(FSS)*<br>Anxiety *(HADS-A)*<br>Depression *(HADS-D)*	Disability *(EDSS, WSAS)*	Stress *(PSS)*		Moderate
**Stroke**								
Radkoll et al. (2022)<br>Kirzinger et al. (2021)<br>Nave et al. (2019)	RCT	n = 200<br>age = 69 ± 12<br>Stroke 5 - 45 days ago<br>BI ≤ 65	PMR (vs exercise)<br>Group<br>25min, 5/week, 4 weeks	Strength *(MRC)*<br>Spasticity *(REPAS)*<br>Depression *(CES-D)*<br>Sleep *(PSQI)*<br>Cognition *(MOCA, TMT, RWT)*<br>Endurance *(spirometry)*<br>Immunological functions *(biomarkers)*<br>Endocrine function *(serum cortisol)*	Walking *(10MWT & 6MWT)*<br>ADL *(BI)*<br>Mobility *(RMI, actigraphy, FAC)*<br>Manual dexterity *(BBT)*<br>Disability (mRS)	Quality of Life *(EQ-5D-5L)*	Safety *(adverse events, falls)*	Moderate
Oostra et al. (2015)	RCT	n = 44<br>age = 52 ± 12<br>Stroke < 1 year ago	PMR (vs motor imagery)<br>Individual<br>30min, 1/day, 6 weeks	Motor Imagination *(MIQ-RS, Walking Trajectory Test)*<br>Sensorimotor impairment *(LE-FMA)*	Walking *(10MWT)*			High

Carin-Levy et al. (2009)	Quali-tative study (follow-up RCT)	n = 14<br>age = 70 ± 10<br>participation in the primary RCT	PMR+DB (vs exercise)<br>Group<br>Unclear, 3/week, 12 weeks				Experiences *(interviews)*	Moderate
Mead et al. (2007)	Pilot RCT	n = 66<br>age = 72 ± 10	PMR+DB (vs exercise)<br>Group<br>20–49min, 3/week, 12 weeks	Health status *(SF36)*<br>Anxiety *(HADS-A)*<br>Depression *(HADS-D)*<br>Leg extensor explosive power *(W/kg)*<br>Exercise tolerance (VO_2 mL/kg/min)	ADLs *(NEADLs)*<br>Mobility *(RMI, functional reach, Sit-to-Stand, TUG, EMS, FAC)*<br>Walking (*m/s*)<br>Independence *(FIM)*		Feasibility	Moderate
**SCI**								
Lovas et al. (2017)	RCT	n = 40<br>age = 46 ± 12<br>SCI > 12month	GI (vs massage)<br>Unclear<br>30min, 1/week, 5 weeks	Pain *(SF-MPQ)*<br>Fatigue *(CFS)*<br>Mood *(POMS)*			Experiences *(comments on intervention)*	Low
Wardell et al. (2016)	Pilot mixed-method trial	12<br>55 ± 8<br>> 6 month post-SCI<br>> 1 month neurogenic pain<br>VAS > 5	GI (vs massage)<br>Unclear<br>30min, 1/week, 5 weeks	Pain (*BPI, VAS)*<br>Mood *(POMS)*<br>Depression *(CES-D)*		Life-Satisfaction *(SWLS)*	Experiences *(interviews)*	Low

Note. Studies are organized by neurological condition and year of publication. Quality ratings are not directly comparable across study designs. MS = Multiple Sclerosis, SCI = Spinal Cord Injury, RCT = Randomized Controlled Trial in parallel design if not further specified, n = number of participants after allocation, age in years ± one standard deviation (SD), rounded to the nearest tenth;

Interventions: ACT = Acceptance and Commitment Therapy, AT = Autogenic Training, CBT = Cognitive Behavioral Therapy, DB = Diaphragmatic Breathing, EMDR = Eye Movement Desensitization and Reprocessing, GI = Guided Imagery, PMR = Progressive Muscle Relaxation, SMOoTh = Self-Management Occupational Therapy Intervention Program, VR = Virtual Reality;

Outcomes: ADLs = Activities of Daily Living, QoL = Quality of Life, PTSD = Post-Traumatic Stress Disorder;

Assessments: AAQ-II = Acceptance and Action Questionnaire, ABC = Activities-Specific Balance Confidence Scale, BBI = Berg Balance Scale, BBT = Box and Block Test, BDI = Beck Depression Inventory, BI = Barthel Index, BPI = Brief Pain Inventory, BRB-N = Brief Repeatable Battery of Neuropsychological Tests, CAPS = Clinician-Administered PTSD Scale, CD-RISC = Connor–Davidson Resilience Scale, CES-D = Center for Epidemiologic Studies Depression Scale, CMDI = Chicago Multiscale Depression Inventory, COPM = Canadian Occupational Performance Measure, DDS = Drexel Defusion Scale, EDSS = Expanded Disability Status Scale, EMS = Elderly Mobility Scale, EQ-5D-5L = European Quality of Life 5 Dimensions, FAC = Functional Ambulation Categories, FAMS = Functional Assessment of Multiple Sclerosis, FGA = Functional Gait Assessment, FIM = Functional Independence Measure, FIS = Fatigue Impact Scale, FSS = Fatigue Severity Scale, HADS- A/D = Hospital Anxiety and Depression Scale – Anxiety/Depression subscale, IES-R = Impact of Event Scale – Revised, LE-FMA = Lower Extremity Fugl–Meyer Assessment, MAAS = Mindful Attention Awareness Scale, MIQ-RS = Movement Imagery Questionnaire – Revised, Second Version, MOCA = Montreal Cognitive Assessment, MPQ = McGill Pain Questionnaire, MSOT = Modified Sensory Organization Test, MSNQ = Multiple Sclerosis Neuropsychological Questionnaire, MSQoL= Multiple Sclerosis Quality of Life, MSWS = Multiple Sclerosis Walking Scale, mRS = Modified Rankin Scale, MRC = Medical Research Council scale for muscle strength, NEADLs = Nottingham Extended Activities of Daily Living, NHPT = Nine-Hole Peg Test, NRS = Numeric Rating Scale, PIS = Pain Interference Scale, POMS = Profile of Mood States, PSQI = Pittsburgh Sleep Quality Index, PSS = Perceived Stress Scale, RMDQ = Roland Morris Disability Questionnaire, REPAS = Resistance to Passive Movement Scale, RMI = Rivermead Mobility Index, RWT = Regensburger Word Fluency Test, SEIQoL-DW = Schedule for the Evaluation of Individual Quality of Life – Direct Weighting, SF-36 = Short Form 36 Health Survey,, SF-MPQ = Short-Form McGill Pain Questionnaire, SHCS = Stanford Hypnotic Clinical Scale, SWLS = Satisfaction With Life Scale, TAQ = Trauma

and Abuse Questionnaire, TES = Treatment Expectancy Scale, TMT = Trail Making Test, TUG = Timed Up and Go, VLQ = Valued Living Questionnaire, VAS = Visual Analogue Scale, VO_2 mL/kg/min = oxygen uptake per kilogram of body weight per minute, W/kg = Watts per kilogram, Walking Trajectory Test = Walking Trajectory Test, WSAS= Work and Social Adjustment Scale, 6MWT = 6 Minute Walk Test, 10MWT = 10 Meter Walk Test;

References

1. Brambila-Tapia AJL, Gutiérrez-García MM, Ruiz-Sandoval JL, Vázquez-Vázquez D, Ramírez-Martínez JF, Macias-Islas MÁ, et al. Using hypnoanalysis and guided imagery to identify and manage emotional aspects of multiple sclerosis. *Explore (NY)* 2022; 18: 88–95. https://doi.org/10.1016/j.explore.2020.10.002
2. Ozkul C, Guclu-Gunduz A, Yazici G, Atalay Guzel N, Irkec C. Effect of immersive virtual reality on balance, mobility, and fatigue in patients with multiple sclerosis: A single-blinded randomized controlled trial. *Eur J Integr Med* 2020; 35: 101092. https://doi.org/10.1016/j.eujim.2020.101092
3. Ozkul C, Guclu-Gunduz A, Eldemir K, Apaydin Y, Gulsen C, Yazici G, et al. Effect of task-oriented circuit training on motor and cognitive performance in patients with multiple sclerosis: A single-blinded randomized controlled trial. *NeuroRehabilitation* 2020; 46: 343–353. https://doi.org/10.3233/NRE-203029
4. Ozkul C, Guclu-Gunduz A, Eldemir K, Apaydin Y, Yazici G, Irkec C. Combined exercise training improves cognitive functions in multiple sclerosis patients with cognitive impairment: A single-blinded randomized controlled trial. *Mult Scler Relat Disord* 2020; 45: 102419. https://doi.org/10.1016/j.msard.2020.102419
5. Giovannetti AM, Quintas R, Tramacere I, Giordano A, Confalonieri P, Messmer Uccelli M, et al. A resilience group training program for people with multiple sclerosis: Results of a pilot single-blind randomized controlled trial and nested qualitative study. *PLoS One* 2020; 15: e0231380. https://doi.org/10.1371/journal.pone.0231380
6. Fox EE, Hough AD, Creanor S, Gear M, Freeman JA. Effects of Pilates-Based Core Stability Training in Ambulant People With Multiple Sclerosis: Multicenter, Assessor-Blinded, Randomized Controlled Trial. *Phys Ther* 2016; 96: 1170–1178. https://doi.org/10.2522/ptj.20150166

7. Carletto S, Borghi M, Bertino G, Oliva F, Cavallo M, Hofmann A, et al. Treating Post-traumatic Stress Disorder in Patients with Multiple Sclerosis: A Randomized Controlled Trial Comparing the Efficacy of Eye Movement Desensitization and Reprocessing and Relaxation Therapy. *Front Psychol* 2016; 7: 526. https://doi.org/10.3389/fpsyg.2016.00526
8. Kos D, Duportail M, Meirte J, Meeus M, D'hooghe MB, Nagels G, et al. The effectiveness of a self-management occupational therapy intervention on activity performance in individuals with multiple sclerosis-related fatigue: a randomized-controlled trial. *Int J Rehabil Res* 2016; 39: 255–262. https://doi.org/10.1097/MRR.0000000000000178
9. Castro-Sánchez AM, Matarán-Peñarrocha GA, Lara-Palomo I, Saavedra-Hernández M, Arroyo-Morales M, Moreno-Lorenzo C. Hydrotherapy for the Treatment of Pain in People with Multiple Sclerosis: A Randomized Controlled Trial. *J Evid Based Complementary Altern Med* 2012; 2012: 1–8. https://doi.org/10.1155/2012/473963
10. Nordin L, Rorsman I. Cognitive behavioural therapy in multiple sclerosis: A randomized controlled pilot study of acceptance and commitment therapy. *J Rehabil Med* 2012; 44: 87–90. https://doi.org/10.2340/16501977-0898
11. Jensen MP, Barber J, Romano JM, Molton IR, Raichle KA, Osborne TL, et al. A Comparison of Self-Hypnosis Versus Progressive Muscle Relaxation in Patients With Multiple Sclerosis and Chronic Pain. *Int J Clin Exp Hypn* 2009; 57: 198–221. https://doi.org/10.1080/00207140802665476
12. Van Kessel K, Moss-Morris R, Willoughby E, Chalder T, Johnson MH, Robinson E. A Randomized Controlled Trial of Cognitive Behavior Therapy for Multiple Sclerosis Fatigue. *Psychosom Med* 2008; 70: 205–213. https://doi.org/10.1097/PSY.0b013e3181643065
13. Kirzinger B, Stroux A, Rackoll T, Endres M, Flöel A, Ebinger M, et al. Elevated Serum Inflammatory Markers in Subacute Stroke Are Associated With Clinical Outcome but Not Modified by Aerobic Fitness Training: Results of the Randomized Controlled PHYS-STROKE Trial. *Front Neurol* 2021; 12: 713018. https://doi.org/10.3389/fneur.2021.713018
14. Rackoll T, Nave AH, Ebinger M, Endres M, Grittner U, Flöel A, et al. Physical Fitness Training in Patients with Subacute Stroke (PHYS-STROKE): Safety analyses of a randomized clinical trial. *Int J Stroke* 2022; 17: 93–100. https://doi.org/10.1177/17474930211006286
15. Nave AH, Rackoll T, Grittner U, Bläsing H, Gorsler A, Nabavi DG, et al. Physical Fitness Training in Patients with Subacute Stroke (PHYS-STROKE): multicentre, randomised controlled, endpoint blinded trial. *BMJ* 2019; 366: l5101. https://doi.org/10.1136/bmj.l5101

16. Oostra K, Oomen A, Vanderstraeten G, Vingerhoets G. Influence of motor imagery training on gait rehabilitation in sub-acute stroke: A randomized controlled trial. *J Rehabil Med* 2015; 47: 204–209. https://doi.org/10.2340/16501977-1908
17. Carin-Levy G, Kendall M, Young A, Mead G. The Psychosocial Effects of Exercise and Relaxation Classes for Persons Surviving a Stroke. *Can J Occup Ther* 2009; 76: 73–80. https://doi.org/10.1177/000841740907600204
18. Mead GE, Greig CA, Cunningham I, Lewis SJ, Dinan S, Saunders DH, et al. Stroke: A Randomized Trial of Exercise or Relaxation. *J Am Geriatr Soc* 2007; 55: 892–899. https://doi.org/10.1111/j.1532-5415.2007.01185.x
19. Lovas J, Tran Y, Middleton J, Bartrop R, Moore N, Craig A. Managing pain and fatigue in people with spinal cord injury: a randomized controlled trial feasibility study examining the efficacy of massage therapy. *Spinal Cord* 2017; 55: 162–166. https://doi.org/10.1038/sc.2016.156
20. Wardell DW, Rintala DH, Duan Z, Tan G. A Pilot Study of Healing Touch and Progressive Relaxation for Chronic Neuropathic Pain in Persons With Spinal Cord Injury. *J Holist Nurs* 2006; 24: 231–240. https://doi.org/10.1177/0898010106289840

Appendix S4

Table SIV: Detailed critical appraisal results

Main Author & Year	Q1	Q2	Q3	Q4	Q5	Q6	Q7	Q8	Q9	Q10	Q11	Q12	Q13	Q14	Q15	Q16	Q17	% Yes	Quality Rating
**Randomized Controlled Trials**																			
Akbari et al. (2020)	Y	U	Y	N	N	N	N	Y	Y	Y	Y	Y	Y	NI	NI	NI	NI	61,5	Moderate
Artemiadis et al. (2012)	Y	N	Y	N	N	Y	N	Y	Y	Y	N	Y	Y	NI	NI	NI	NI	61,5	Moderate
Beitollahi et al. (2022)	Y	U	N	N	N	N	N	Y	Y	Y	N	Y	Y	NI	NI	NI	NI	46,2	Low
Brambila-Tapia et al. (2022)	N	Y	Y	N	N	N	Y	Y	Y	Y	N	Y	N	NI	NI	NI	NI	53,8	Low
Carletto et al. (2017)	Y	Y	Y	N	N	Y	Y	Y	Y	N	N	Y	Y	NI	NI	NI	NI	69,2	Moderate
Case et al. (2018)	N	U	N	N	N	N	Y	Y	Y	Y	N	Y	Y	NI	NI	NI	NI	46,2	Low
Castro-Sánchez et al. (2012)	Y	Y	Y	N	N	Y	Y	Y	Y	Y	N	Y	Y	NI	NI	NI	NI	76,9	Moderate
Chang et al. (2020)	Y	Y	Y	N	N	Y	Y	Y	Y	Y	N	Y	Y	NI	NI	NI	NI	76,9	Moderate
Fox et al. (2016)	Y	Y	Y	N	N	N	Y	Y	Y	Y	N	Y	Y	NI	NI	NI	NI	69,2	Moderate
Garis et al. (2023)	Y	Y	Y	N	N	Y	U	Y	Y	Y	Y	Y	Y	NI	NI	NI	NI	76,9	Moderate
Golding et al. (2016)	Y	Y	Y	N	N	N	N	Y	Y	Y	N	Y	Y	NI	NI	NI	NI	61,4	Moderate
Kesik et al. (2022)	Y	U	Y	N	N	N	U	Y	Y	Y	N	Y	Y	NI	NI	NI	NI	53,8	Low
Kos et al. (2016)	Y	Y	Y	N	N	Y	Y	Y	Y	Y	Y	Y	Y	NI	NI	NI	NI	84,6	High
Lovas et al. (2017)	U	U	Y	N	N	Y	N	Y	Y	U	U	Y	Y	NI	NI	NI	NI	46,2	Low
Mackay et al. (2015)	Y	N	Y	N	N	Y	N	Y	Y	Y	Y	Y	Y	NI	NI	NI	NI	69,2	Moderate

Main Author & Year	Q1	Q2	Q3	Q4	Q5	Q6	Q7	Q8	Q9	Q10	Q11	Q12	Q13	Q14	Q15	Q16	Q17	% Yes	Quality Rating
Mackereth et al. (2009)	Y	Y	N	N	N	Y	U	Y	Y	Y	N	Y	N	NI	NI	NI	NI	53,8	Low
Masoudi et al. (2013)	U	U	Y	N	N	N	N	Y	Y	Y	Y	Y	Y	NI	NI	NI	NI	53,8	Low
Mead et al. (2007)	Y	U	Y	N	N	Y	U	Y	Y	Y	Y	Y	Y	NI	NI	NI	NI	69,2	Moderate
Mirhosseini et al. (2019)	Y	U	Y	N	N	N	N	Y	Y	U	U	Y	Y	NI	NI	NI	NI	46,2	Low
Mirhosseini et al. (2021)	Y	U	Y	N	N	N	N	Y	Y	Y	Y	Y	Y	NI	NI	NI	NI	53,8	Low
Mohd Nordin et al. (2025)	Y	U	Y	N	N	Y	Y	Y	Y	Y	Y	Y	Y	NI	NI	NI	NI	76,9	Moderate
Monden et al. (2024)	Y	U	Y	N	N	Y	Y	Y	Y	Y	Y	Y	Y	NI	NI	NI	NI	76,9	Moderate
Nave et al. (2019)	Y	U	N	N	N	Y	Y	Y	Y	Y	Y	Y	Y	NI	NI	NI	NI	69,2	Moderate
Nazari et al. (2018)	Y	U	Y	N	N	N	Y	Y	Y	N	N	Y	Y	NI	NI	NI	NI	53,8	Low
Nordin and Rorsman (2012)	Y	U	Y	N	N	Y	N	Y	Y	Y	Y	Y	Y	NI	NI	NI	NI	69,2	Moderate
Novais et al. (2016)	Y	U	Y	N	N	N	N	N	N	Y	U	Y	Y	NI	NI	NI	NI	38,5	Low
Oostra et al. (2015)	Y	Y	Y	N	N	Y	Y	Y	Y	Y	Y	Y	Y	NI	NI	NI	NI	84,6	High
Ozkul et al. (2020a)	Y	U	Y	N	N	N	Y	Y	Y	N	N	Y	Y	NI	NI	NI	NI	53,8	Low
Ozkul et al. (2020b)	Y	U	Y	N	N	N	Y	Y	Y	Y	N	Y	Y	NI	NI	NI	NI	61,5	Moderate
Ozkul et al. (2020c)	Y	U	Y	N	N	N	Y	Y	Y	Y	Y	Y	Y	NI	NI	NI	NI	69,2	Moderate
Safi (2015)	U	U	Y	N	N	N	N	Y	Y	Y	Y	Y	Y	NI	NI	NI	NI	53,8	Low
Sutherland et al. (2005)	U	U	N	N	N	N	N	Y	Y	N	N	Y	N	NI	NI	NI	NI	23,1	Low
van Kessel et al. (2008)	Y	Y	N	N	N	Y	N	Y	Y	Y	Y	Y	Y	NI	NI	NI	NI	69,2	Moderate

Main Author & Year	Q1	Q2	Q3	Q4	Q5	Q6	Q7	Q8	Q9	Q10	Q11	Q12	Q13	Q14	Q15	Q16	Q17	% Yes	Quality Rating
**Quasi-Experimental Studies**																			
Dayapoğlu and Tan (2012)	Y	N	Y	Y	N	Y	Y	N	Y	NI	NI	NI	NI	NI	NI	NI	NI	66,7	Moderate
Ghafari et al. (2009)	Y	Y	Y	N	Y	Y	Y	N	Y	NI	NI	NI	NI	NI	NI	NI	NI	77,8	Moderate
Hampson et al. (2020)	Y	N	Y	Y	Y	Y	Y	N	Y	NI	NI	NI	NI	NI	NI	NI	NI	87,5	High
Javdan et al. (2021)	Y	Y	U	N	Y	Y	U	U	Y	NI	NI	NI	NI	NI	NI	NI	NI	55,6	Low
Jensen et al. (2009)	Y	Y	Y	Y	Y	Y	U	N	Y	NI	NI	NI	NI	NI	NI	NI	NI	77,8	Moderate
Kneebone et al. (2014)	Y	N	Y	Y	Y	Y	U	Y	Y	NI	NI	NI	NI	NI	NI	NI	NI	77,8	Moderate
Rohrmann et al. (2001)	Y	Y	Y	Y	N	Y	N	Y	Y	NI	NI	NI	NI	NI	NI	NI	NI	77,8	Moderate
Saifan et al. (2021)	Y	Y	Y	N	N	Y	Y	Y	Y	NI	NI	NI	NI	NI	NI	NI	NI	77,8	Moderate
Schlesinger et al. (2014)	Y	N	Y	Y	Y	Y	N	Y	Y	NI	NI	NI	NI	NI	NI	NI	NI	77,8	Moderate
Wang et al. (2024)	Y	Y	Y	N	Y	Y	U	Y	Y	NI	NI	NI	NI	NI	NI	NI	NI	77,8	Moderate
Wood et al. (2021)	Y	N	Y	Y	N	Y	U	N	Y	NI	NI	NI	NI	NI	NI	NI	NI	55,6	Low
**Case Series**																			
El-Helou, et al. (2024)	Y	Y	Y	N	N	Y	Y	Y	NA	Y	NI	NI	NI	NI	NI	NI	NI	77,8	Moderate
Grzesiak (1977)	N	N	N	U	U	Y	Y	N	N	N	NI	NI	NI	NI	NI	NI	NI	20	Low
Mauersberger et al. (2000)	N	Y	Y	U	U	Y	N	N	N	Y	NI	NI	NI	NI	NI	NI	NI	40	Low
Moriya and Ikeda (2013)	Y	Y	Y	N	U	N	N	Y	N	Y	NI	NI	NI	NI	NI	NI	NI	50	Low
Ortega (1978)	N	Y	N	U	U	N	N	Y	Y	Y	NI	NI	NI	NI	NI	NI	NI	40	Low

Main Author & Year	Q1	Q2	Q3	Q4	Q5	Q6	Q7	Q8	Q9	Q10	Q11	Q12	Q13	Q14	Q15	Q16	Q17	% Yes	Quality Rating
Park et al. (2025)	Y	Y	Y	U	U	N	Y	Y	N	Y	NI	NI	NI	NI	NI	NI	NI	60	Moderate
Schoffl et al. (2023)	Y	Y	Y	N	N	N	Y	Y	Y	Y	NI	NI	NI	NI	NI	NI	NI	70	Moderate
**Case Reports**																			
De Luca et al. (2018)	Y	N	N	Y	Y	Y	N	Y	NI	NI	NI	NI	NI	NI	NI	NI	NI	62,5	Moderate
**Qualitative Studies**																			
Carin-Levy et al. (2009)	U	Y	Y	Y	Y	N	U	Y	Y	Y	NI	NI	NI	NI	NI	NI	NI	70	Moderate
El-Helou et al. (2023)	U	Y	Y	Y	Y	N	Y	Y	Y	Y	NI	NI	NI	NI	NI	NI	NI	80	High
**Mixed-method Studies**																			
Giovannetti et al. (2016)	Y	Y	Y	Y	Y	Y	Y	Y	N	N	N	Y	Y	Y	Y	Y	N	76,5	Moderate
Ostojic et al. (2022)	Y	Y	Y	Y	Y	Y	Y	N	Y	N	N	Y	Y	Y	N	N	N	64,7	Moderate
Wang et al. (2022)	Y	Y	Y	Y	Y	N	Y	Y	N	Y	N	Y	Y	N	Y	N	N	64,7	Moderate
Wang et al. (2019)	Y	Y	Y	N	Y	Y	Y	N	Y	Y	Y	Y	Y	Y	Y	Y	Y	88,2	High
Wardell et al. (2016)	Y	Y	Y	N	N	Y	Y	N	N	Y	N	Y	Y	N	N	N	N	47,1	Low

Note. Item-level critical appraisal ratings are presented for all included studies. Different appraisal tools were applied according to study design; therefore, appraisal items are displayed as standardized columns Q1 to Q17, with full item wording provided in the item legend below. Ratings were coded as Y = yes, N = no, U = unclear, NA = not applicable, NI = item not included in the respective tool. The percentage of yes ratings was calculated using applicable items only and excluding NA and NI ratings. Automated quality ratings were derived descriptively from the proportion of yes ratings (>80% High, 60-80% Moderate and >60% Low). Quality ratings are not directly comparable across study designs.

Item Legend:

Randomized controlled trials (60): Q1: Was true randomization used for assignment of participants to treatment groups? Q2: Was allocation to treatment groups concealed? Q3: Were treatment groups similar at the baseline? Q4: Were participants blind to treatment assignment? Q5: Were those delivering treatment blind to treatment assignment? Q6: Were outcomes assessors blind to treatment assignment? Q7: Were treatment groups treated identically other than the intervention of interest? Q8: Was follow up complete and if not, were differences between groups in terms of their follow up adequately described and analyzed? Q9: Were participants analyzed in the groups to which they were randomized? Q10: Were outcomes measured in the same way for treatment groups?

Q11: Were outcomes measured in a reliable way? Q12: Was appropriate statistical analysis used? Q13: Was the trial design appropriate, and any deviations from the standard RCT design (individual randomization, parallel groups) accounted for in the conduct and analysis of the trial*?*

Quasi-experimental studies (61): Q1: Is it clear in the study what is the "cause" and what is the "effect"? Q2: Was there a control group? Q3: Were participants included in any comparisons similar? Q4: Were the participants included in any comparisons receiving similar treatment/care, other than the exposure or intervention of interest? Q5: Were there multiple measurements of the outcome both pre and post the intervention/exposure? Q6: Were the outcomes of participants included in any comparisons measured in the same way? Q7: Were outcomes measured in a reliable way? Q8: Was follow-up complete and, if not, were differences between groups in terms of their follow-up adequately described and analyzed? Q9: Was appropriate statistical analysis used?

Case series (62): Q1: Were there clear criteria for inclusion in the case series? Q2: Was the condition measured in a standard, reliable way for all participants included in the case series? Q3: Were valid methods used for identification of the condition for all participants included in the case series? Q4: Did the case series have consecutive inclusion of participants? Q5: Did the case series have complete inclusion of participants? Q&: Was there clear reporting of the demographics of the participants included in the study? Q7: Was there clear reporting of clinical information of the participants? Q8: Were the outcomes or follow-up results of cases clearly reported? Q9: Was there clear reporting of the presenting sites'/clinics' demographic information? Q10: Was statistical analysis appropriate?

Case report (63): Q1: Were patient's demographic characteristics clearly described? Q2: Was the patient's history clearly described and presented as a timeline? Q3: Was the current clinical condition of the patient on presentation clearly described? Q4: Were diagnostic tests or assessment methods and the results clearly described? Q5: Was the intervention(s) or treatment procedure(s) clearly described? Q6: Was the post-intervention clinical condition clearly described? Q7: Were adverse events (harms) or unanticipated events identified and described? Q8: Does the case report provide takeaway lessons?

Qualitative studies (64): Q1: Is there congruity between the philosophical or theoretical positioning of the study and the methodological approach that has been adopted Q2: Is there congruity between the research methodology and the research question or objectives? Q3: Is there congruity between the research methodology and the methods used to collect data? Q4: Is there congruity between the research methodology and the representation and analysis of data? Q5: Is there congruity between the research methodology and the interpretation of results? Q6: Is there a statement locating the researcher culturally or theoretically? Q7: Is the influence of the researcher on the research, and vice- versa, addressed? Q8: Are participants, and where appropriate, their voices, adequately represented? Q9: Is the research ethical according to current criteria, and for recent studies, is there evidence of ethical approval by an appropriate body? Q10: Do the conclusions drawn in the research report flow from the analysis, or interpretation, of the data?

Mixed-method studies (65): Q1: Are there clear research questions? Q2: Do the collected data allow to address the research questions? Q3: Is the qualitative approach appropriate to answer the research question? Q4: Are the qualitative data collection methods adequate to address the research question? Q5: Are the findings adequately derived from the data? Q6: Is the interpretation of results sufficiently substantiated by data? Q7: Is there coherence between qualitative data sources, collection, analysis and interpretation? Q8: Is the sampling strategy relevant to address the research question? Q9: Is the sample representative of the target population? Q10: Are the measurements appropriate? Q11: Is the risk of nonresponse bias low? Q12: Is the statistical analysis appropriate to answer the research question? Q13: Is there an adequate rationale for using a mixed methods design to address the research question? Q14: Are the different components of the study effectively integrated to answer the research question? Q15: Are the outputs of the integration of qualitative and quantitative components adequately interpreted? Q16: Are divergences and inconsistencies between quantitative and qualitative results adequately addressed? Q17: Do the different components of the study adhere to the quality criteria of each tradition of the methods involved?

References

1. Akbari A, Ahmadi F, Jalili E, Khazaei S. The Effect of Relaxation Technique (Jacobsen and Benson) on Depression, Anxiety, and Stress in Patients with Multiple Sclerosis. *Curr Psychiatry Res Rev* 2021; 16: 213–219. https://doi.org/10.2174/2666082216999200819105928

2. Artemiadis AK, Vervainioti AA, Alexopoulos EC, Rombos A, Anagnostouli MC, Darviri C. Stress Management and Multiple Sclerosis: A Randomized Controlled Trial. *Arch Clin Neuropsychol* 2012; 27: 406–416. https://doi.org/10.1093/arclin/acs039
3. Beitollahi M, Forouzi MA, Tirgari B, Jahani Y. Fatigue, stigma, and mood in patients with multiple sclerosis: effectiveness of guided imagery. *BMC Neurol* 2022; 22: 152. https://doi.org/10.1186/s12883-022-02677-3
4. Brambila-Tapia AJL, Gutiérrez-García MM, Ruiz-Sandoval JL, Vázquez-Vázquez D, Ramírez-Martínez JF, Macias-Islas MÁ, et al. Using hypnoanalysis and guided imagery to identify and manage emotional aspects of multiple sclerosis. *Explore (NY)* 2022; 18: 88–95. https://doi.org/10.1016/j.explore.2020.10.002
5. Carletto S, Borghi M, Bertino G, Oliva F, Cavallo M, Hofmann A, et al. Treating Post-traumatic Stress Disorder in Patients with Multiple Sclerosis: A Randomized Controlled Trial Comparing the Efficacy of Eye Movement Desensitization and Reprocessing and Relaxation Therapy. *Front Psychol* 2016; 7: 526. https://doi.org/10.3389/fpsyg.2016.00526
6. Case LK, Jackson P, Kinkel R, Mills PJ. Guided Imagery Improves Mood, Fatigue, and Quality of Life in Individuals With Multiple Sclerosis: An Exploratory Efficacy Trial of Healing Light Guided Imagery. *J Evid Based Complementary Altern Med* 2018; 23: 2515690X17748744. https://doi.org/10.1177/2515690X17748744
7. Castro-Sánchez AM, Matarán-Peñarrocha GA, Lara-Palomo I, Saavedra-Hernández M, Arroyo-Morales M, Moreno-Lorenzo C. Hydrotherapy for the Treatment of Pain in People with Multiple Sclerosis: A Randomized Controlled Trial. *J Evid Based Complementary Altern Med* 2012; 2012: 1–8. https://doi.org/10.1155/2012/473963
8. Chang W-L, Lee J-T, Li C-R, Davis AHT, Yang C-C, Chen Y-J. Effects of Heart Rate Variability Biofeedback in Patients With Acute Ischemic Stroke: A Randomized Controlled Trial. *Biol Res Nurs* 2020; 22: 34–44. https://doi.org/10.1177/1099800419881210
9. Fox EE, Hough AD, Creanor S, Gear M, Freeman JA. Effects of Pilates-Based Core Stability Training in Ambulant People With Multiple Sclerosis: Multicenter, Assessor-Blinded, Randomized Controlled Trial. *Phys Ther* 2016; 96: 1170–1178. https://doi.org/10.2522/ptj.20150166
10. Garis G, Dettmers C, Hildebrandt A, Duning T, Hildebrandt H. Comparing two relaxation procedures to ease fatigue in multiple sclerosis: a single-blind randomized controlled trial. *Neurol Sci* 2023; 44: 4087–4098. https://doi.org/10.1007/s10072-023-07042-x

11. Golding K, Kneebone I, Fife-Schaw C. Self-help relaxation for post-stroke anxiety: a randomised, controlled pilot study. *Clin Rehabil* 2016; 30: 174–180. https://doi.org/10.1177/0269215515575746
12. Kesik G, Ozdemir L, Mungan Ozturk S. The Effects of Relaxation Techniques on Pain, Fatigue, and Kinesiophobia in Multiple Sclerosis Patients: A 3-Arm Randomized Trial. *J Neurosci Nurs* 2022; 54: 86–91. https://doi.org/10.1097/JNN.0000000000000620
13. Kos D, Duportail M, Meirte J, Meeus M, D'hooghe MB, Nagels G, et al. The effectiveness of a self-management occupational therapy intervention on activity performance in individuals with multiple sclerosis-related fatigue: a randomized-controlled trial. *Int J Rehabil Res* 2016; 39: 255–262. https://doi.org/10.1097/MRR.0000000000000178
14. Lovas J, Tran Y, Middleton J, Bartrop R, Moore N, Craig A. Managing pain and fatigue in people with spinal cord injury: a randomized controlled trial feasibility study examining the efficacy of massage therapy. *Spinal Cord* 2017; 55: 162–166. https://doi.org/10.1038/sc.2016.156
15. Mackay AM, Buckingham R, Schwartz RS, Hodgkinson S, Beran RG, Cordato DJ. The Effect of Biofeedback as a Psychological Intervention in Multiple Sclerosis: A Randomized Controlled Study. *Int J MS Care* 2015; 17: 101–108. https://doi.org/10.7224/1537-2073.2014-006
16. Mackereth PA, Booth K, Hillier VF, Caress A-L. Reflexology and progressive muscle relaxation training for people with multiple sclerosis: A crossover trial. *Complement Ther Clin Pract* 2009; 15: 14–21. https://doi.org/10.1016/j.ctcp.2008.07.002
17. Masoudi R, Sharifi Faradonbeh A, Mobasheri M, Moghadasi J. Evaluating the Effectiveness of Using a Progressive Muscle Relaxation Technique in Reducing the Pain of Multiple Sclerosis Patients. *J Musculoskelet Pain* 2013; 21: 350–357. https://doi.org/10.3109/10582452.2013.852150
28. Mead GE, Greig CA, Cunningham I, Lewis SJ, Dinan S, Saunders DH, et al. Stroke: A Randomized Trial of Exercise or Relaxation. *J Am Geriatr Soc* 2007; 55: 892–899. https://doi.org/10.1111/j.1532-5415.2007.01185.x
19. Mirhosseini S, Mohammadi A, Rezaei M, Mirbagher Ajorpaz N. The Effect of Benson Relaxation Technique on the Fatigue Severity of Patients With MS. *JCCNC* 2019; 5: 175–182. https://doi.org/10.32598/JCCNC.5.3.175
20. Mirhosseini S, Rezaei M, Ajorpaz NM. The Effect of Benson Relaxation Technique on General Health in Multiple Sclerosis (MS) Patients in Kashan, Iran: A Randomized Controlled Trial. *J Res Dev Nurs Midw* 2021; 18: 17–20. https://doi.org/10.29252/jgbfnm.18.1.17

21. Mohd Nordin NA, Thazhakkattu Vasu D, Ezzat Ghazali S. Effectiveness of Autogenic Relaxation Training as an Adjunct to Usual Physiotherapy in Enhancing Emotional Well-Being and Functional Independence in Stroke Survivors: A Randomized Controlled Study. *Top Stroke Rehabil* 2025; 32: 573–585. https://doi.org/10.1080/10749357.2024.2444117
22. Monden KR, Nupp J, Ali A, MacIntyre B, Sevigny M, Hanks Philippus A. Exploring the feasibility of heart rate variability biofeedback for individuals with tetraplegia: A pilot clinical trial. *Rehabil Psychol* 2024; 70: 214–225. https://doi.org/10.1037/rep0000577
23. Nave AH, Rackoll T, Grittner U, Bläsing H, Gorsler A, Nabavi DG, et al. Physical Fitness Training in Patients with Subacute Stroke (PHYS-STROKE): multicentre, randomised controlled, endpoint blinded trial. *BMJ* 2019; 366: l5101. https://doi.org/10.1136/bmj.l5101
24. Nazari F, Soheili M, Hosseini S, Shaygannejad V. A comparison of the effects of reflexology and relaxation on pain in women with multiple sclerosis. *J Complement Integr Med* 2016; 13: 65–71. https://doi.org/10.1515/jcim-2015-0046
25. Nordin L, Rorsman I. Cognitive behavioural therapy in multiple sclerosis: A randomized controlled pilot study of acceptance and commitment therapy. *J Rehabil Med* 2012; 44: 87–90. https://doi.org/10.2340/16501977-0898
26. Novais PGN, Batista KDM, Grazziano EDS, Amorim MHC. The effects of progressive muscular relaxation as a nursing procedure used for those who suffer from stress due to multiple sclerosis. *Rev Lat Am Enfermagem* 2016; 24: e2789. https://doi.org/10.1590/1518-8345.1257.2789
27. Oostra K, Oomen A, Vanderstraeten G, Vingerhoets G. Influence of motor imagery training on gait rehabilitation in sub-acute stroke: A randomized controlled trial. *J Rehabil Med* 2015; 47: 204–209. https://doi.org/10.2340/16501977-1908
28. Ozkul C, Guclu-Gunduz A, Yazici G, Atalay Guzel N, Irkec C. Effect of immersive virtual reality on balance, mobility, and fatigue in patients with multiple sclerosis: A single-blinded randomized controlled trial. *Eur J Integr Med* 2020; 35: 101092. https://doi.org/10.1016/j.eujim.2020.101092
29. Ozkul C, Guclu-Gunduz A, Eldemir K, Apaydin Y, Gulsen C, Yazici G, et al. Effect of task-oriented circuit training on motor and cognitive performance in patients with multiple sclerosis: A single-blinded randomized controlled trial. *NeuroRehabilitation* 2020; 46: 343–353. https://doi.org/10.3233/NRE-203029

30. Ozkul C, Guclu-Gunduz A, Eldemir K, Apaydin Y, Yazici G, Irkec C. Combined exercise training improves cognitive functions in multiple sclerosis patients with cognitive impairment: A single-blinded randomized controlled trial. *Mult Scler Relat Disord* 2020; 45: 102419. https://doi.org/10.1016/j.msard.2020.102419
31. Safi SZ. A Fresh Look at the Potential Mechanisms of Progressive Muscle Relaxation Therapy on Depression in Female Patients With Multiple Sclerosis. *Iran J Psychiatry Behav Sci* 2015; 9: e340. https://doi.org/10.17795/ijpbs340
32. Sutherland G, Andersen MB, Morris T. Relaxation and Health-Related Quality of Life in Multiple Sclerosis: The Example of Autogenic Training. *J Behav Med* 2005; 28: 249–256. https://doi.org/10.1007/s10865-005-4661-2
33. Van Kessel K, Moss-Morris R, Willoughby E, Chalder T, Johnson MH, Robinson E. A Randomized Controlled Trial of Cognitive Behavior Therapy for Multiple Sclerosis Fatigue. *Psychosom Med* 2008; 70: 205–213. https://doi.org/10.1097/PSY.0b013e3181643065
34. Dayapoğlu N, Tan M. Evaluation of the Effect of Progressive Relaxation Exercises on Fatigue and Sleep Quality in Patients with Multiple Sclerosis. *J Altern Complement Med* 2012; 18: 983–987. https://doi.org/10.1089/acm.2011.0390
35. Ghafari S, Ahmadi F, Nabavi M, Anoshirvan K, Memarian R, Rafatbakhsh M. Effectiveness of applying progressive muscle relaxation technique on quality of life of patients with multiple sclerosis. *J Clin Nurs* 2009; 18: 2171–2179. https://doi.org/10.1111/j.1365-2702.2009.02787.x
36. Hampson N, King L, Eriksson L-M, Smee H. The effects of relaxation training on depression and anxiety in people living with long-term neurological conditions. *Disabil Rehabil* 2020; 42: 2100–2105. https://doi.org/10.1080/09638288.2018.1554009
37. Javdan T, Imani E, Negahi AA. Evaluation the effect of progressive muscle relaxation technique on fatigue and daily living activities in patients with Multiple Sclerosis. *PJMHS* 2021; 15: 1773–1777. https://doi.org/10.53350/pjmhs211561773
38. Jensen MP, Barber J, Romano JM, Molton IR, Raichle KA, Osborne TL, et al. A Comparison of Self-Hypnosis Versus Progressive Muscle Relaxation in Patients With Multiple Sclerosis and Chronic Pain. *Int J Clin Exp Hypn* 2009; 57: 198–221. https://doi.org/10.1080/00207140802665476
39. Kneebone II, Van Zanden BE, Dorstyn DS, Roberts RM, Lord SR, Querstret D, et al. Relaxation and related therapies for people with multiple sclerosis (MS): A systematic review. *Clin Rehabil* 2022; 36: 883–899. https://doi.org/10.1177/02692155221091509

40. Rohrmann S, Hopf M, Hennig J, Netter P. Psychobiologische Effekte von Autogenem Training und Progressiver Muskelrelaxation bei Patienten mit Rückenschmerzen, Patienten mit Multipler Sklerose und Gesunden. *ZPPP* 2001; 49: 373–387.
41. Saifan AR, Aburuz ME, Dhaher EA, Rayyan A, Jaberi MA, Masa'Deh R. The Effect of Benson Relaxation Technique on Depression, Anxiety, and Stress of Jordanian Patients Diagnosed with Multiple Sclerosis: A Cross-Sectional Study. *Depress Res Treat* 2021; 2021: 1–8. https://doi.org/10.1155/2021/8300497
42. Schlesinger I, Benyakov O, Erikh I. Relaxation Guided Imagery Reduces Motor Fluctuations in Parkinson's Disease. *J Parkinsons Dis* 2014; 4: 431–436. https://doi.org/10.3233/JPD-130338
43. Wang Y-L, Wu W-X, Yang C-C, Huang S-M, Chang C-C, Li C-R, et al. Heart rate variability biofeedback enhances cognitive, motor, psychological, and autonomic functions in post-stroke rehabilitation. *Int J Psychophysiol* 2024; 203: 112411. https://doi.org/10.1016/j.ijpsycho.2024.112411
44. Wood C, Cutshall SM, Lawson DK, Ochtrup HM, Henning NB, Larsen BE, et al. Music Therapy for Anxiety and Pain After Spinal Cord Injury: A Pilot Study. *Glob Adv Health Med* 2021; 10: 21649561211058697. https://doi.org/10.1177/21649561211058697
45. El-Helou R, Ryan B, Hilari K, Kneebone I. Evaluating the co-designed "kalmer" relaxation intervention for people with aphasia: a feasibility case series. *Aphasiology* 2024; 1–18. https://doi.org/10.1080/02687038.2024.2437784
46. Grzesiak RC. Relaxation techniques in treatment of chronic pain. *Arch Phys Med Rehabil* 1977; 58: 270–272.
47. Mauersberger K, Artz K, Duncan B, Gurgevich S. Can children with spastic cerebral palsy use self-hypnosis to reduce muscle tone? A preliminary study. *Int Med* 2000; 2: 93–96. https://doi.org/10.1016/S1096-2190(00)00008-1
48. Moriya R, Ikeda N. A pilot study of the effects of progressive muscle relaxation on fatigue specific to multiple sclerosis. *Br J Neurosci Nurs* 2013; 9: 35–41. https://doi.org/10.12968/bjnn.2013.9.1.35
49. Ortega DF. Relaxation Exercise with Cerebral Palsied Adults Showing Spasticity. *J Appl Behav Anal* 1978; 11: 447–451. https://doi.org/10.1901/jaba.1978.11-447
50. Park J, Jung M, Ha J, Park JJ, Im S. Smartphone-based non-invasive biofeedback therapy for post-stroke sleep disorders: short report. *Front Neurol* 2025; 16: 1601821. https://doi.org/10.3389/fneur.2025.1601821

51. Schoffl J, Arora M, Pozzato I, McBain C, Rodrigues D, Vafa E, et al. Heart Rate Variability Biofeedback in Adults with a Spinal Cord Injury: A Laboratory Framework and Case Series. *J Clin Med* 2023; 12: 7664. https://doi.org/10.3390/jcm12247664
52. De Luca R, Torrisi M, Piccolo A, Bonfiglio G, Tomasello P, Naro A, et al. Improving post-stroke cognitive and behavioral abnormalities by using virtual reality: A case report on a novel use of nirvana. *Appl Neuropsychol Adult* 2018; 25: 581–585. https://doi.org/10.1080/23279095.2017.1338571
53. Carin-Levy G, Kendall M, Young A, Mead G. The Psychosocial Effects of Exercise and Relaxation Classes for Persons Surviving a Stroke. *Can J Occup Ther* 2009; 76: 73–80. https://doi.org/10.1177/000841740907600204
54. El-Helou R, Ryan B, Kneebone I. Development of the *"Kalmer"* relaxation intervention: co-design with stroke survivors with aphasia. *Disabil Rehabil* 2023; 45: 1517–1529. https://doi.org/10.1080/09638288.2022.2069294
55. Giovannetti AM, Quintas R, Tramacere I, Giordano A, Confalonieri P, Messmer Uccelli M, et al. A resilience group training program for people with multiple sclerosis: Results of a pilot single-blind randomized controlled trial and nested qualitative study. *PLoS One* 2020; 15: e0231380. https://doi.org/10.1371/journal.pone.0231380
56. Ostojic K, Sharp N, Paget S, Khut G, Morrow A. BrightHearts: A pilot study of biofeedback assisted relaxation training for the management of chronic pain in children with cerebral palsy. *Paediatr Neonatal Pain* 2022; 4: 33–42. https://doi.org/10.1002/pne2.12062
57. Wang X, Thiel L, Graff ND. Mindfulness and Relaxation Techniques for Stroke Survivors with Aphasia: A Feasibility and Acceptability Study. *Healthcare* 2022; 10: 1409. https://doi.org/10.3390/healthcare10081409
58. Wang X, Smith C, Ashley L, Hyland ME. Tailoring Self-Help Mindfulness and Relaxation Techniques for Stroke Survivors: Examining Preferences, Feasibility and Acceptability. *Front Psychol* 2019; 10: 391. https://doi.org/10.3389/fpsyg.2019.00391
59. Wardell DW, Rintala DH, Duan Z, Tan G. A Pilot Study of Healing Touch and Progressive Relaxation for Chronic Neuropathic Pain in Persons With Spinal Cord Injury. *J Holist Nurs* 2006; 24: 231–240. https://doi.org/10.1177/0898010106289840
60. Barker TH, Stone JC, Sears K, Klugar M, Tufanaru C, Leonardi-Bee J, et al. The revised JBI critical appraisal tool for the assessment of risk of bias for randomized controlled trials. *JBI Evid Synth* 2023; 21: 494–506. https://doi.org/10.11124/JBIES-22-00430

61. Barker TH, Habibi N, Aromataris E, Stone JC, Leonardi-Bee J, Sears K, et al. The revised JBI critical appraisal tool for the assessment of risk of bias for quasi-experimental studies. *JBI Evid Synth* 2024; 22: 378–388. https://doi.org/10.11124/JBIES-23-00268
62. Munn Z, Barker TH, Moola S, Tufanaru C, Stern C, McArthur A, et al. Methodological quality of case series studies: an introduction to the JBI critical appraisal tool. *JBI Evid Synth* 2020; 18: 2127–2133. https://doi.org/10.11124/JBISRIR-D-19-00099
63. Moola S, Munn Z, Tufanaru C, Aromataris E, Sears K, Sfetcu R, et al. Systematic reviews of etiology and risk. In: Aromataris E, Munn Z, eds. JBI Manual for Evidence Synthesis. JBI.
64. Porritt K, Evans C, Bennett C, Loveday H, Bjerrum M, Salmond S, et al. Systematic reviews of qualitative evidence. In: Aromataris E, Lockwood C, Porritt K, Pilla B, Jordan Z, eds. JBI Manual for Evidence Synthesis. JBI.
65. Hong QN, Fàbregues S, Bartlett G, Boardman F, Cargo M, Dagenais P, et al. The Mixed Methods Appraisal Tool (MMAT) version 2018 for information professionals and researchers. *Education for Information* 2018; 34: 285–291. https://doi.org/10.3233/EFI-180221
